# Single-cell analysis of breast cancer metastasis reveals epithelial-mesenchymal plasticity signatures associated with poor outcomes

**DOI:** 10.1172/JCI164227

**Published:** 2024-09-03

**Authors:** Juliane Winkler, Weilun Tan, Catherine M.M. Diadhiou, Christopher S. McGinnis, Aamna Abbasi, Saad Hasnain, Sophia Durney, Elena Atamaniuc, Daphne Superville, Leena Awni, Joyce V. Lee, Johanna H. Hinrichs, Patrick S. Wagner, Namrata Singh, Marco Y. Hein, Michael Borja, Angela M. Detweiler, Su-Yang Liu, Ankitha Nanjaraj, Vaishnavi Sitarama, Hope S. Rugo, Norma Neff, Zev J. Gartner, Angela Oliveira Pisco, Andrei Goga, Spyros Darmanis, Zena Werb

**Affiliations:** 1 Department of Anatomy and; 2 Department of Cell and Tissue Biology, UCSF, San Francisco, California, USA.; 3 Center for Cancer Research, Medical University of Vienna, Vienna, Austria.; 4 Chan Zuckerberg Biohub SF, San Francisco, California, USA.; 5 Department of Pharmaceutical Chemistry, UCSF, San Francisco, California, USA.; 6 Institute of Internal Medicine D, Medical Cell Biology, University Hospital Münster, Münster, Germany.; 7 Max Perutz Labs, Vienna Biocenter Campus (VBC), Vienna, Austria.; 8 Medical University of Vienna, Max Perutz Labs, Vienna, Austria.; 9 Helen Diller Family Comprehensive Cancer Center, UCSF, San Francisco, California, USA.; 10 Chan Zuckerberg Biohub Investigator, San Francisco, California, USA.; 11 Genentech, South San Francisco, California, USA.

**Keywords:** Oncology, Bioinformatics, Breast cancer

## Abstract

Metastasis is the leading cause of cancer-related deaths. It is unclear how intratumor heterogeneity (ITH) contributes to metastasis and how metastatic cells adapt to distant tissue environments. The study of these adaptations is challenged by the limited access to patient material and a lack of experimental models that appropriately recapitulate ITH. To investigate metastatic cell adaptations and the contribution of ITH to metastasis, we analyzed single-cell transcriptomes of matched primary tumors and metastases from patient-derived xenograft models of breast cancer. We found profound transcriptional differences between the primary tumor and metastatic cells. Primary tumors upregulated several metabolic genes, whereas motility pathway genes were upregulated in micrometastases, and stress response signaling was upregulated during progression. Additionally, we identified primary tumor gene signatures that were associated with increased metastatic potential and correlated with patient outcomes. Immune-regulatory control pathways were enriched in poorly metastatic primary tumors, whereas genes involved in epithelial-mesenchymal transition were upregulated in highly metastatic tumors. We found that ITH was dominated by epithelial-mesenchymal plasticity (EMP), which presented as a dynamic continuum with intermediate EMP cell states characterized by specific genes such as *CRYAB* and *S100A2*. Elevated expression of an intermediate EMP signature correlated with worse patient outcomes. Our findings identified inhibition of the intermediate EMP cell state as a potential therapeutic target to block metastasis.

## Abstract 2

Profiles of primary breast cancer tumors matched at single-cell resolution with metastases reveal features of intratumoral heterogeneity that contribute to metastasis.

## Introduction

Metastases account for the vast majority of cancer-related deaths (1), however, why some cancers metastasize while others do not is poorly understood. Specific genetic alterations do not define metastatic progression (2), suggesting that metastasis is controlled by the capability of individual tumor cells to phenotypically adapt to different microenvironments that are distinct from their site of origin. However, it is unclear how intratumor heterogeneity (ITH) affects the underlying processes that lead to these adaptations during the multistep process of metastasis.

One concept aimed at explaining these complex phenotypic changes is that tumor cells undergo epithelial-mesenchymal transition (EMT) and acquire mesenchymal features, allowing the dissemination of tumor cells to distant organs (3). However, to form overt metastasis, disseminated tumor cells need to regain epithelial features by undergoing mesenchymal-epithelial transition (MET). While numerous studies have established a role for EMT in cancer progression, few have examined the role of the bidirectional dynamic process of epithelial-mesenchymal plasticity (EMP) (4, 5). EMT is often defined by the loss or gain of a few canonical markers that are involved in cell adhesion and motility (e.g., *VIM*, *EPCAM*, *CDH1*, and *CDH2*) and whose expression is regulated by a set of core transcription factors (TFs) (e.g., *SNAI1*, *SNAI2*, *TWIST1*, and *ZEB1*) (6). However, the expression of these commonly used markers is context and tissue dependent and can change dynamically during the EMP process (7–9). Moreover, tumor tissues are heterogeneous, and various phenotypes and cell states may occur within a single tumor. To better understand the contributions of EMP to the metastatic process, a comprehensive analysis of individual tumor cells at both the primary tumor and metastatic site is needed.

Breast cancer (BC) is a genetically and phenotypically highly diverse disease that is categorized into different molecular subtypes and clinically evaluated on the basis of genetic and molecular features that are associated with patient outcomes and guide treatment strategies. However, there is a high degree of heterogeneity within these classifications with regard to cellular phenotypes and their distribution between and within patients (10), which may result in therapy failure, and, ultimately, approximately 30% of patients with BC die from metastases (11). Studies addressing the contributions of BC ITH in metastasis are lacking (12).

Advances in single-cell transcriptomics have enabled the study of ITH in BC (13–16) and other cancers (17, 18). Some recent studies have investigated metastasis (13, 14, 19, 20), however, these studies either lacked matched primary tumor tissues for comparison, included only small numbers of samples, or lacked distant metastatic samples beyond the local draining lymph nodes (19–23). Analyzing metastases is technically difficult because they may consist of individual or small numbers of cells within complex tissues, which are difficult to locate and isolate from patients. To overcome some of these limitations, we used patient-derived xenograft (PDX) models, as they represent an ideal model to study the effect of ITH on metastasis. These models preserve the heterogeneity of the human tumor, spontaneously metastasize, and thereby recapitulate every step in the metastatic cascade when orthotopically transplanted and mimic the metastatic pattern in the patient (24–28).

To investigate the contributions of ITH and EMP to metastasis, we performed single-cell RNA-Seq (scRNA-Seq) of a large panel of PDX models of human BC that retained ITH and had diverse spontaneous metastatic potential. We used 2 scRNA-Seq approaches: one to capture ITH (high-throughput, droplet-based MULTI-Seq) and one to capture rare metastatic cells (low-throughput, plate-based Smart-Seq2), yielding a rich and comprehensive data set that allowed us to identify transcriptional differences between primary tumors, their matched metastases, and gene signatures associated with metastatic potential. We found that primary tumors and metastasis had strong transcriptional differences. Moreover, we found that highly metastatic tumors had elevated expression of EMT markers and that EMP was a key feature of ITH within both the primary tumor and at the site of metastasis. Along the EMP continuum, we identified intermediate EMP cell states characterized by specific marker genes. Elevated expression of EMP marker genes was associated with worse outcomes for patients with BC.

## Results

### BC PDX models possess varying metastatic potential.

**Figure 1:**
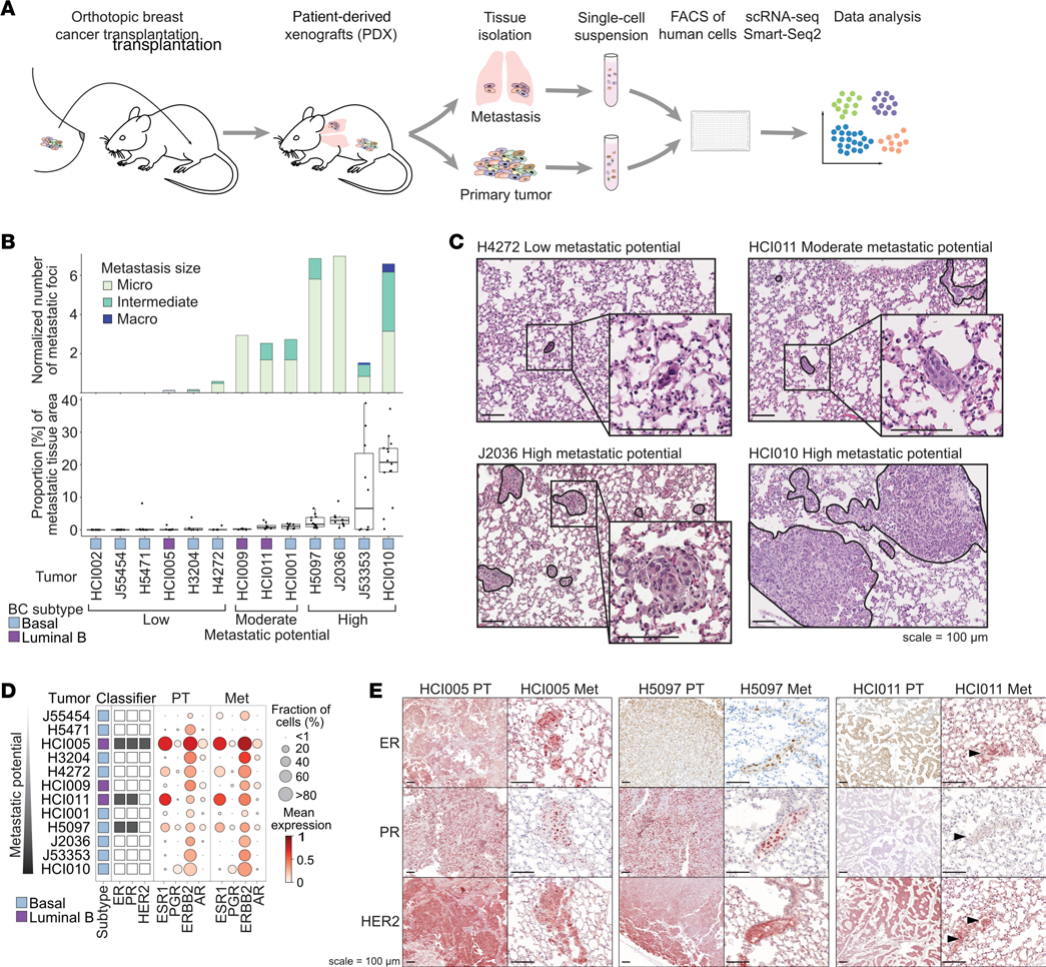
BC PDX models show varying metastatic potential. (**A**) Experimental overview: Lung metastases and primary tumor tissues were isolated from orthotopically transplanted BC PDX models and dissociated. The resulting single-cell suspensions were FACS enriched for human cells using a human specific antibody (hCD298) and sorted into 384-well plates (1 cell per well), and scRNA-Seq was performed using Smart-Seq2. Data analysis investigated tumor heterogeneity and differences between primary tumor and metastatic cells. (**B**) Bar chart shows the median number of metastatic foci per mm^2^ lung tissue area per model (upper panel), determined by histology. Metastatic foci were classified as micrometastasis (< 10 cells), intermediate (10–100 cells), and macrometastasis (>100 cells). Box plot shows the fraction of metastatic tissue per total lung tissue area, determined by histology (lower panel). The *x*-axis shows the model, BC subtype, and metastatic potential. (**C**) Representative H&E-stained images of metastatic lung tissue for low, moderate, and high metastatic potential models. Scale bars: 100 μm. (**D**) Bubble plot showing the expression of receptors in primary tumor (PT) and metastatic cells (Met) per model. (**E**) Representative images showing immunohistochemical staining for ER, PR, and HER2 in primary tumors and metastatic lungs of ER^+^ tumor models. Arrowheads indicate metastasis. When possible, the same metastasis is shown in consecutive sections. Scale bars: 100 μm.

We characterized the metastatic phenotype of PDX models derived from 13 patients with BC belonging to different BC subtypes (3 luminal B, 10 basal). Our PDXs included 2 estrogen receptor^+^ (ER^+^) and progesterone receptor^+^ (PR^+^) models; 1 triple-positive (ER, PR, human epidermal growth factor receptor 2 [HER2]) model; and 10 triple-negative BC (TNBC) models (24, 26, 27, 29). Three of the basal TNBC PDX models were newly established in this study (Supplemental Table 1; supplemental material available online with this article; https://doi.org/10.1172/JCI164227DS1). Human breast tumors were orthotopically transplanted into the cleared mouse mammary fat pad of NOD/SCIDγ mice and spontaneously metastasized to the lungs and other organs. The number and size of the metastatic foci in the lungs were quantified by histology, and the metastatic burden was validated by flow cytometry (Figure 1, B and C, and Supplemental Figure 1, A–C). We chose to focus our efforts on lung metastases because approximately 50% of metastases in patients with BC are found in the lung (30).

Notably, the PDX models showed a reproducible and consistent tendency to metastasize. Therefore, the tumor models were defined as having low (*n* = 6), moderate (*n* = 3), or high metastatic potential (*n* = 4). Models with low metastatic potential exhibited no or very few micrometastases (<10 cells). Models in the moderate category showed more micrometastases and intermediate-sized metastases (10–100 cells), and those with high metastatic potential developed either a high number of micrometastases and/or many macrometastases (>100 cells), resulting in a high metastatic burden (Figure 1, B and C, and Supplemental Figure 1, A–C).

To investigate whether the metastatic potential of each PDX model was a result of aggressive primary tumor growth, we measured the time until each primary tumor reached the endpoint. Surprisingly, we found that the metastatic potential was independent of the primary tumor growth rate (Supplemental Figure 1D). To validate this independence, we performed resection experiments on the HCI002 model, which is fast-growing but poorly metastatic. Tumor resection was followed by tumor recurrence, and metastases were quantified after recurrent tumors reached the endpoint (Supplemental Figure 2, E–H). However, even after primary tumor resection and subsequent tumor recurrence, HCI002 tumor still developed very few, albeit larger, metastatic foci compared with nonresected HCI002 tumors (Supplemental Figure 1F). Quantification of metastasis of resected HCI002 was performed at a time to endpoint similar to that of the slower-growing HCI010 model, which showed very high metastatic potential (Supplemental Figure 1, D and G). These data indicate that the low metastatic potential of HCI002 was independent of its high primary tumor growth rate and suggest that metastasis was not determined by proliferation of the primary tumor.

After having established our metastatic models, we next investigated the transcriptional profiles of primary tumor and metastatic cells. To this end, individual tumor cells were isolated from primary tumors and matched lung metastases from 12 PDX models for scRNA-Seq (Smart-Seq2, Figure 1A). High-quality, single-cell transcriptome data were collected from 2,090 cells (*n* = 1,395 primary tumor; *n* = 695 metastatic cells). As expected, we were unable to isolate any metastatic cells from the poorly metastatic HCI002 model. To benchmark our data set, we determined the PAM50 BC subtype using pseudobulk gene expression and receptor status. The clinically defined PAM50 BC subtype and receptor status were confirmed in most samples according to PAM50 and *ESR1* (ER), *PGR* (PR), and *ERBB2* (HER2) gene expression (Supplemental Table 1 and Figure 1D). Interestingly, *ERBB2* transcripts were detected in all tumors, including those that were not clinically classified as HER2^+^. This may have been attributable to the threshold required for the clinical classification of the original tumor by histochemistry and/or single-region sampling of the heterogeneous original tumor. The observed transcriptional expression of these receptors was validated by immunohistochemistry on the protein level. In addition, receptor expression was maintained in the metastatic cells on the transcript and protein levels (Figure 1, D and E, and Supplemental Figure 1, I and J).

Altogether, we established and characterized PDX models of different BC subtypes with varying metastatic potential independent of their primary tumor growth rate. We found that receptor status was maintained between the primary tumor and metastatic cells.

### Differential gene expression analysis reveals metastasis-associated gene signatures and inter- and intratumoral transcriptional heterogeneity.

**Figure 2:**
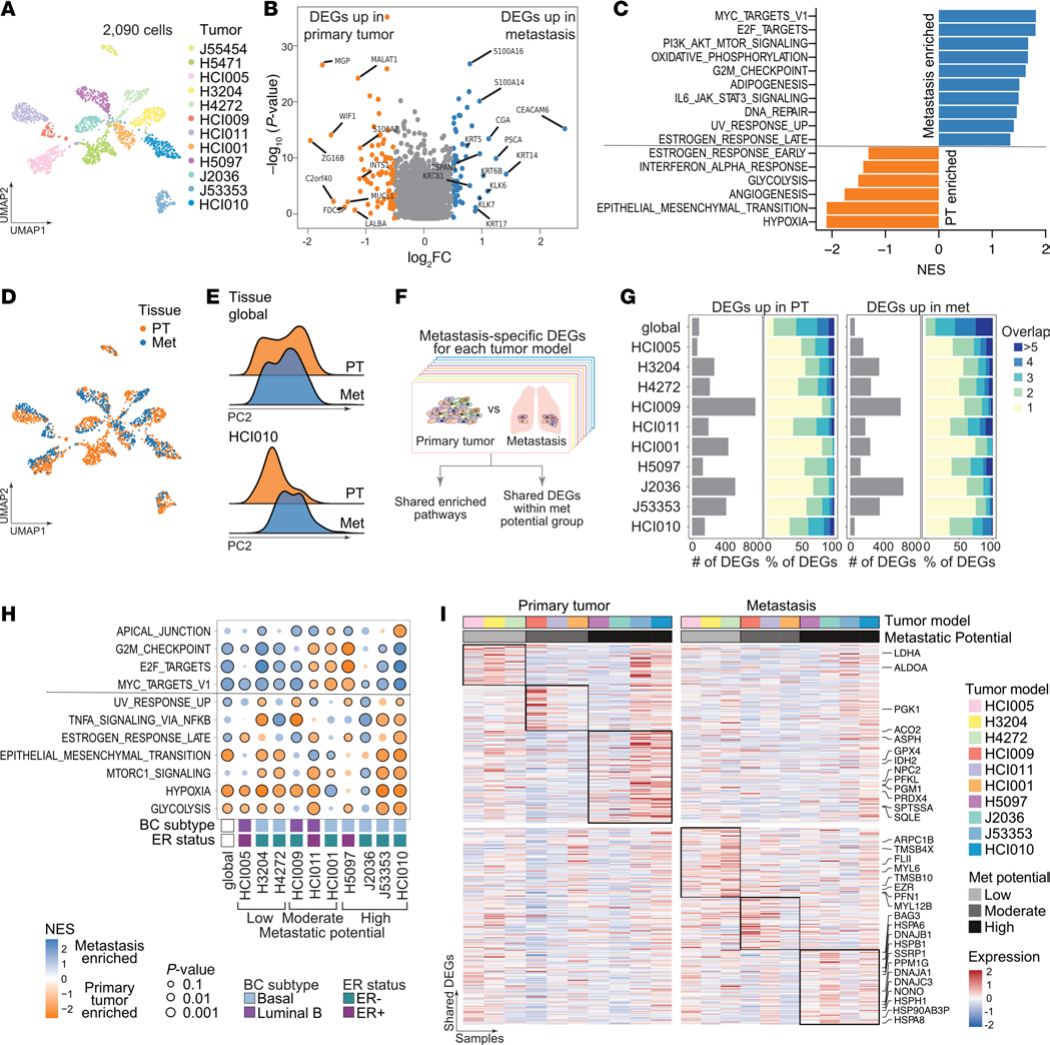
Differential gene expression between primary tumor and matched metastatic cells. (**A**) UMAP projection of single-cell transcriptomes color coded by individual models. (**B**) Volcano plot showing the log_2_-fold expression change and *P* value of DEGs in primary tumors versus metastases using the MAST test. The top 10 DEGs are highlighted (orange = upregulated [up] in primary tumor, blue = up in metastases). (**C**) Bar plot showing pathways enriched in DEGs between primary tumors (negative normalized enrichment score [NES], orange) and metastases (positive NES, blue) using HALLMARK gene sets (MSigDB). (**D**) UMAP projection of single-cell transcriptomes color coded by primary tumor (orange) and metastasis (blue). (**E**) Ridge plots showing normalized cell counts along PC2 in primary tumors and metastases for all models grouped (global, upper panel) and a representative individual model (HCI010, lower panel). (**F**) Workflow for identification of metastasis-specific DEGs in each model. (**G**) Bar charts showing the number of DEGs (gray bars) upregulated in primary tumors (left) and metastases (right) for each model. Color bars indicate the proportion of upregulated DEGs that are shared between 2 or several models (blue color scale) or exclusive to 1 model (yellow). (**H**) Bubble plot showing enriched HALLMARK pathways (MSigDB) obtained using DEGs between individual primary tumors and matched metastases that are shared among at least 3 tumors. (**I**) Heatmaps showing the mean expression of upregulated DEGs between the primary tumor (left) or metastases (right) in individual models that were shared between at least 2 models within the same metastatic potential (black box).

Before investigating the transcriptional heterogeneity in primary and metastatic cells, we first interrogated the major sources of variation in our data set using principal component (PC) analysis. This analysis illustrated that cells were separating along PC1 based on the ER status and BC subtype of the tumor model (Supplemental Figure 2, A and B), indicating that these features were major sources of variation in the data set. Moreover, individual tumors clustered separately from other tumors, reflecting the effect of interpatient heterogeneity on gene expression (Figure 2A). Notably, we did not detect variability between technical batches (individual plates) or biological replicates (same tumor implanted into different animals) (Supplemental Figure 2, C and D).

Next, we sought to identify genes unique to metastatic cells shared across tumor models. To this end, cells were grouped across all samples by tissue (primary tumor or metastasis), and differential gene expression was evaluated using Model-based Analysis of Single Cell Transcriptomics (MAST) (31) with the tumor model as a covariate (Supplemental Figure 2E). We found 132 differentially expressed genes (DEGs), 79 of which were upregulated in metastatic cells and conserved across all 12 tumor models (log_2_ fold change >0.5, *P* < 0.05; Figure 2B, Supplemental Figure 2F, and Supplemental Table 2). Among the top metastasis-associated genes were several cytokeratins (*KRT5*, *KRT6B*, *KRT14*, *KRT17*, *KRT81*), calcium-binding S100 proteins (*S100A16*, *S100A14*), heat shock protein HSP1, cell-surface proteins such as *TSPAN1*, serine proteases (*KLK6*, *KLK7*), and the glycoproteins carcinoembryonic antigen–related cell adhesion molecule 6 (*CEACAM6*) and prostate stem cell antigen (*PSCA*).

Gene set enrichment analysis revealed that metastatic cells were enriched in pathways of c-MYC, E2F, and PI3K/AKT/MTOR signaling and oxidative phosphorylation (Figure 2C). Interestingly, metastatic cells also upregulated genes involved in protumor survival and immune-suppressive pathways (IL6/JAK/STAT3, Figure 2C and Supplemental Figure 2G) (32). In contrast, hypoxia, EMT, angiogenesis, and glycolysis were enriched in primary tumor cells (Figure 2C). However, we observed a profound inter- and intratumoral heterogeneity of the expression of DEGs associated with the top enriched pathways in either primary tumor (hypoxia) or metastatic cells (MYC) (Supplemental Figure 2, H and I). Interestingly, within individual models, primary tumor and metastatic cells showed strong transcriptional differences, as illustrated by their distinct clustering (Figure 2D) and by their separation along PC2 when analyzed individually (Figure 2E and Supplemental Figure 2, J and N).

Excited by the observation that primary and metastatic cells showed distinct transcriptional landscapes on an individual tumor level, and to account for the pronounced variability among tumor models, we next analyzed DEGs between primary tumor and metastatic cells for each model separately (Supplemental Table 2) and compared the identified DEGs across tumors (Figure 2F). Given the insufficient numbers of metastatic cells (<10), 2 tumor models with low metastatic potential (J55454, H5471) were excluded from this analysis (Supplemental Figure 2, K–M). The different models showed a wide range of numbers of upregulated genes in primary tumor and metastatic cells (Figure 2G). Notably, approximately 50% of the upregulated genes in each tissue were tumor model specific, and only a few (<5%) were shared among more than 5 models, again highlighting the magnitude of interpatient heterogeneity (Figure 2G). To determine whether transcriptional differences could be related to the genetic relationships between primary tumor and metastatic cells, we compared their copy number variation (CNV) profiles. This analysis revealed a strong correlation (*R^2^* = 0.744–0.971) between inferred CNV profiles of primary tumors and their matched metastases, suggesting that their genetic profile was very similar and might only have a small effect on the transcriptional differences (Supplemental Figure 2O).

Next, we evaluated the enriched pathways in primary tumors or metastases that were shared among tumor models. Although most shared pathways were also identified in the previous analysis of all models combined (Figure 2C and Supplemental Figure 2G), some pathways showed intriguing differences in enrichment among tumor models. For example, while the combined analysis revealed an overall suppression of the estrogen response pathway in the primary tumor, the individual analyses showed that this pathway was specifically enriched in ER^+^ primary tumors (HCI005, HCI011, H5097) compared with the matched metastatic samples (Figure 2H). This suggests that estrogen signaling is impaired in metastatic cells despite the maintenance of ESR1 expression (Figure 1, D and E, and Supplemental Figure 1I). Additionally, although this analysis indicated that metastatic cells from some models showed enrichment in the G_2_M checkpoint pathway, we could not confirm the occurrence of generally more active proliferation or substantial shifts in the cell cycle of metastatic cells (Supplemental Figure 2, P and Q). The enrichment of glycolysis and hypoxia seemed to be a general feature of primary tumors; this was not surprising, as primary tumors have limited access to nutrients when they grow in size. Moreover, EMT was enriched in either primary tumors or metastases in the majority of the analyzed models, indicating the dynamic activity of this pathway in both compartments.

To identify shared features relevant to the metastatic phenotype, we identified DEGs that were common among models with a similar metastatic potential. We found 74 genes upregulated in metastatic cells that were shared between at least 2 tumors with low metastatic potential (Figure 2I and Supplemental Table 3). Among these, many genes were involved in cytoskeleton assembly and cell motility (e.g., *MYL12B*, *MYL6*, *PFN1*, *TMSB4X*, *TMSB10*, *ARPC1B*, *EZR*, *FLII*). In contrast, among the 91 genes upregulated in metastatic cells from highly metastatic tumor models, many genes were indicative of high stress-response signaling, including several heat shock proteins (*HSPB1*, *HSPA8*, *HSPA6*, *HSPH1*, *HSP90AB3P*, *DnaJs A1*, *B1*, *C3*, and *BAG3*), *PPM1G*, and genes involved in DNA damage repair (*SSRP1*, *NONO*). Genes involved in glycolysis (*ALDOA*, *LDHA*, *PGK1*, *PFKL*, *PGM1*) and other metabolic processes (*GPX4*, *PRDX4*, *ACO2*, *ASPH*, *IDH2*, *SQLE*, *NPC2*, *SPTSSA*) were upregulated in primary tumor cells, suggesting that the metabolism in primary tumors was distinct from that in their matched metastasis.

In summary, we observed strong transcriptional differences between primary tumor and metastatic cells at the individual tumor level, with a majority of tumor-specific DEGs. Shared features across models were the upregulation of hypoxia, glycolysis, and other metabolism-related genes in primary tumors. The shared upregulated genes among metastatic cells of poorly metastatic tumor models were involved in cytoskeleton assembly and motility. Stress-response signaling was increased in metastases from the highly metastatic models.

### Metastatic signatures are correlated with patient outcomes.

**Figure 3:**
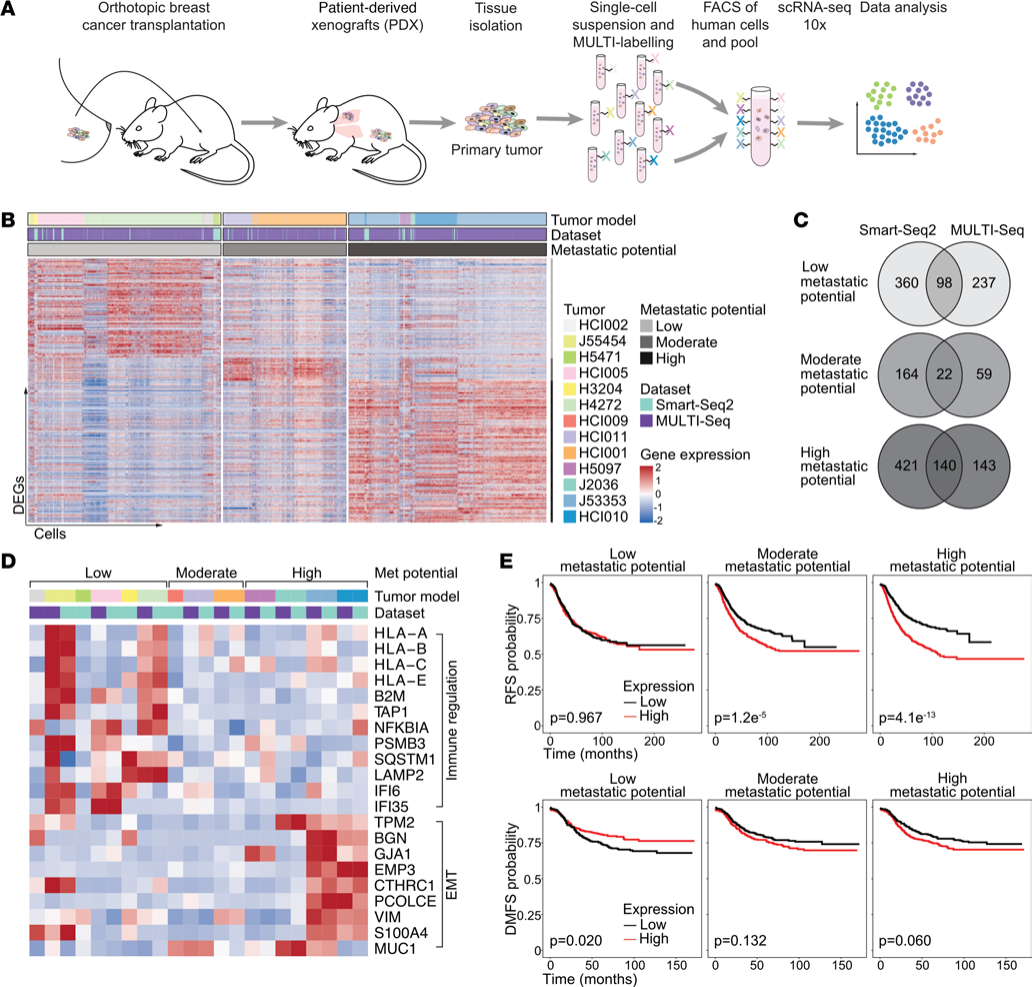
Metastatic signatures are correlated with patient outcomes. (**A**) Schematic workflow of the MULTI-Seq experimental setup. (**B**) Heatmap showing expression of DEGs between individual tumors and tumors of the other metastatic potential groups that are shared between at least 2 tumors. (**C**) Venn diagram showing the number of DEGs shared between the Smart-Seq2 and MULTI-Seq data sets for the different metastatic potential groups. (**D**) Heatmap showing the mean expression of selected metastasis-associated genes per tumor model using the same annotations as in Figure 3B. (**E**) Kaplan-Meier plots showing RFS (top, *n* = 2,032 patients) and DMFS (bottom, *n* = 958 patients) of patients with BC using the mean expression of the metastasis-associated gene signatures (generated with KM-plotter) (42). The *P* values using the log-rank test are shown.

After having identified transcriptional differences between primary tumor and metastatic cells that were shared among models with a similar metastatic phenotype, we wondered whether the primary tumor-intrinsic features are correlated with the consistently observed metastatic potential of these tumors. To address this question, we generated an additional, larger scRNA-Seq data set that better reflected the ITH of the primary tumors. To this end, we performed high-throughput, droplet-based scRNA-Seq with MULTI-Seq (33) sample multiplexing on 10 different primary tumors with varying metastatic potential (Figure 3A), generating transcriptomics data from a total of 16,861 tumor cells (Supplemental Figure 3, B–D).

We aimed to identify signatures that were associated with the metastatic potential of the primary tumor while preserving intertumoral heterogeneity. We first identified DEGs that were upregulated in individual tumors when compared with tumors of different metastatic potential groups (Supplemental Table 4 and Supplemental Table 5). We then selected genes that were shared among individual tumors of the same metastatic potential group and identified by both scRNA-Seq methods used in this study (Figure 3, B and C, Supplemental Figure 3A, and Supplemental Table 6). Among the shared genes upregulated in primary tumors with low metastatic potential were those related to immune regulation processes such as antigen processing and cross-presentation (e.g., *HLA-A*, *HLA-B*, *HLA-C*, *HLA-E*, *B2M*, *TAP1*) and innate immunity (e.g., *NFKBIA*, *PSMB3*, *SQSTM1*, *LAMP2*, *IFI6*, *IFI35*) (Figure 3D and Supplemental Figure 3, E and F), which seems surprising, since we were using in vivo models that lacked lymphoid cells. Our findings may be a result of preacquired and maintained selection and/or may reflect tumor-intrinsic, antimetastatic features of these genes. Furthermore, 21 upregulated DEGs were shared among the moderate metastatic potential tumors. Among those were genes involved in stress (heat shock proteins *HSP90AA1* and *HSPD1*) and actin signaling (*CLDN3*, *CLDN7*, *ARPC5L*) and the proinflammatory *HMGB1* gene, which can have pro- and antimetastatic functions (34, 35) (Supplemental Table 6). Genes upregulated in highly metastatic primary tumors included known metastasis-related genes such as *S100A4* (36, 37) and *MUC1* (38), and genes associated with EMT (*VIM*, *PLOD1*, *BGN*) (Figure 3D). MYC signaling was among the top 5 pathways enriched in highly metastatic primary tumors (Supplemental Figure 3G). MYC signaling can suppress IFN signaling and antigen presentation pathways including the downregulation of B2M and MHC-I (39, 40). This inverse correlation could explain the observed enrichment of immune regulatory pathways in poorly metastatic primary tumors compared with highly metastatic tumors, which showed elevated MYC signaling (Supplemental Figure 3H). To test if proliferation affects aggressive progression and metastases we assessed the cell-cycle distribution and proliferation capacity of tumor cells. Neither the proliferation rate nor the cell-cycle phase distributions were significantly different among primary tumors with different metastatic potential (Supplemental Figure 3, I and J), which is in line with our experimental data (Supplemental Figure 1D), indicating that a highly metastatic phenotype was not the result of increased tumor cell proliferation.

Next, to test the clinical implications of our findings, we correlated the observed metastasis-associated signatures with patient-related outcomes by examining publicly available bulk gene expression data from BC patients with different subtypes (41). Indeed, patients with high expression of the genes involved in the poorly metastatic signature exhibited improved distant metastasis–free survival (DMFS) (Figure 3E). High expression of moderate metastatic genes was associated with worse recurrence-free survival (RFS), and high expression of the highly metastatic signature was associated with the worst patient outcomes.

In summary, we identified intrinsic metastasis-associated gene signatures in primary tumors that correlated with patient-related outcomes in a BC subtype–specific manner (Supplemental Figure 4). While genes upregulated in poorly metastatic primary tumors are involved in immune regulation, genes present in the highly metastatic signature were associated with EMT.

### EMP is a key feature of ITH and is associated with metastatic potential.

Markers of EMT were upregulated in primary tumors of highly metastatic tumors as compared with those of low metastatic potential. However, we also found that EMT markers were enriched in either primary tumor or metastatic cells in different models indicating that EMT is a dynamic process during metastatic progression. Next, we sought to classify EMP cell states in heterogeneous tumor cell populations and investigate their effect on metastatic potential in vivo. To this end, we evaluated the expression of a pan-cancer gene signature of 303 mesenchymal and epithelial markers in our data set (42). Canonical epithelial markers (*EPCAM*, *CDH1*) were highly expressed in cells with a high epithelial signature, and mesenchymal markers (*VIM*, *FN1*, *CDH2*) showed the expected expression patterns (Supplemental Figure 5A). However, commonly used EMT markers, such as *FN1* and *CDH2*, were only detected at low levels in some cells with otherwise high levels of mesenchymal markers.

**Figure 4:**
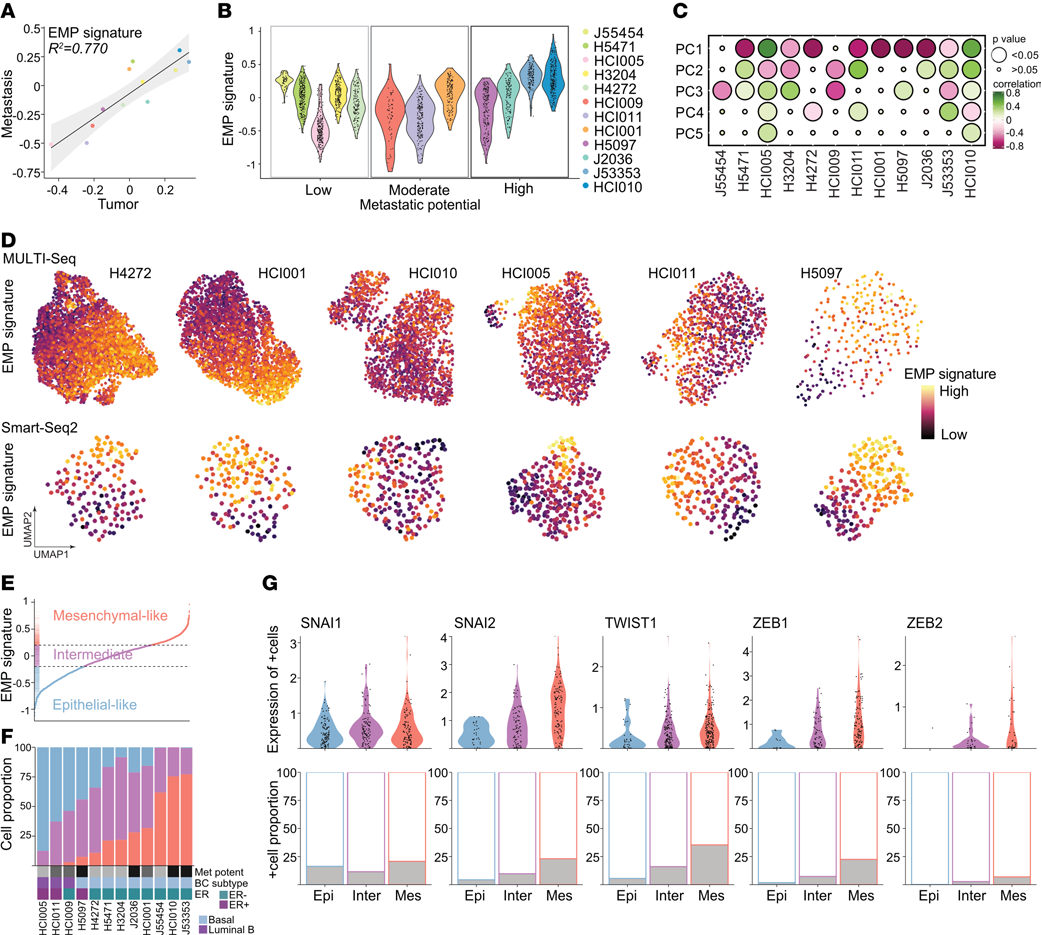
EMP is a key feature of tumor heterogeneity. (**A**) Scatter plot showing the correlation of the mean EMP signature gene expression of the primary tumor and metastatic cells colored by tumor. Linear regression with 95% CIs and Pearson’s correlation coefficient are shown. (**B**) Violin plot showing the EMP signature per tumor ordered by metastatic potential using the Smart-Seq2 data set. (**C**) Bubble plot showing the correlation of the EMP signature with PCs 1–5 using the Smart-Seq2 data set. (**D**) UMAP projections of single-cell transcriptomes for individual tumors are color coded by the magnitude of EMP signature gene expression. (**E**) Cells in the Smart-Seq2 data set ranked by the EMP signature exhibited 3 cell states: epithelial-like (blue), intermediate EMP (purple), and mesenchymal-like cells (red). (**F**) Bar chart showing the proportion of EMP cell states in each tumor ranked by the increasing proportion of mesenchymal-like cells. Grayscale boxes indicate the metastatic potential. Other annotations indicate ER status and BC subtype. The Smart-Seq2 data set is shown. (**G**) Violin plots show the expression of EMT-associated TFs in cells expressing these TFs, grouped by EMP cell state (Epi, epithelial-like; Inter, intermediate EMP, Mes, mesenchymal-like cells). Bar charts show the fraction of TF-expressing cells colored in gray. The Smart-Seq2 data set is shown.

To illustrate the dynamic EMP process, we combined epithelial and mesenchymal signature scores to define the overall EMP cell state; an EMP signature of greater than 0 reflects cells with a more mesenchymal than epithelial signature. The EMP signatures of individual tumor models were strongly correlated (*R^2^* = 0.780) between our 2 data sets (Smart-Seq2 and MULTI-Seq), demonstrating reproducibility across different sequencing methods and experimental replicates (Supplemental Figure 5B). Surprisingly, the average EMP signature was similar for both the primary tumors and their matching metastases (Figure 4A), suggesting an intrinsic determinant of EMP that may be independent of environmental influences. In contrast, the overall EMP signature of each tumor was associated with its metastatic potential (Figure 4B, Smart-Seq2, *R* = 0.336; Supplemental Figure 5C, MULTI-Seq, *R* = 0.606). Moreover, the EMP state was highly variable across cells within a tumor (Figure 4B and Supplemental Figure 5C). Indeed, the EMP signatures of tumors were correlated with the PC1 coordinates, indicating that the EMP cell state was a major source of variation among cells within a tumor and significantly contributed to ITH (Figure 4C and Supplemental Figure 5D). Finally, we observed that the EMP state changed gradually in transcriptional space (UMAP projection), further illustrating that EMP is a continuum of cell states (Figure 4D).

Next, we investigated whether ITH concerning EMP status was associated with metastatic potential. Individual cells were classified into 1 of 3 different EMP cell states according to the magnitude of their EMP signature expression: epithelial-like (< –0.2), intermediate EMP (–0.2–0.2), and mesenchymal-like cells (>0.2) (Figure 4E and Supplemental Figure 5E). ER^+^ and luminal B classified tumors (HCI005, HCI011, HCI009, H5097) showed the highest proportion of epithelial-like cells (Figure 4F and Supplemental Figure 5F). Within this group, the proportion of mesenchymal-like cells was associated with an increased metastatic potential. Similar associations were observed in the group of TNBC basal tumors, which showed an overall higher fraction of mesenchymal-like cells. In TNBC basal tumors, the proportion of mesenchymal-like cells increased with higher metastatic potential with almost no epithelial-like cells present in the tumors with high metastatic potential, although 2 tumor models might not follow this pattern (J55454 and HCI002). Overall, these data indicate that the distribution of the EMP cell states is associated with metastatic potential and may be influenced by BC subtype and/or receptor status (Figure 4F and Supplemental Figure 5F).

Studies suggest that both mesenchymal and epithelial functions are necessary for the metastatic cascade (43). Intermediate EMP cells may have epithelial and mesenchymal capabilities and a high degree of plasticity and therefore might represent cells that are more likely to metastasize (44, 45). However, the intermediate EMP cells, which expressed both epithelial and mesenchymal signatures at similar levels (Figure 4E), were present in every tumor, and their abundance did not correlate with metastatic potential (Figure 4F). Intermediate EMP cells expressed core TFs promoting EMT such as *SNAI2*, *TWIST1*, *ZEB1*, and *ZEB2*(4) at higher levels than epithelial-like cells but lower levels than mesenchymal-like cells, highlighting their intermediate character (upper panels of Figure 4G and Supplemental Figure 5G). Moreover, the fraction of cells expressing these TFs also increased from epithelial-like to intermediate EMP to mesenchymal-like cells (lower panels of Figure 4G and Supplemental Figure 5G).

Collectively, these findings suggest that EMP is a key feature of ITH and associated with metastatic potential. We identified cells coexpressing epithelial and mesenchymal markers that belong to an intermediate EMP cell state. These intermediate EMP cells were present in primary tumors and metastases in all tumor models studied and were characterized by low expression of EMT-associated TFs.

### Intermediate EMP cells express distinct marker genes.

**Figure 5:**
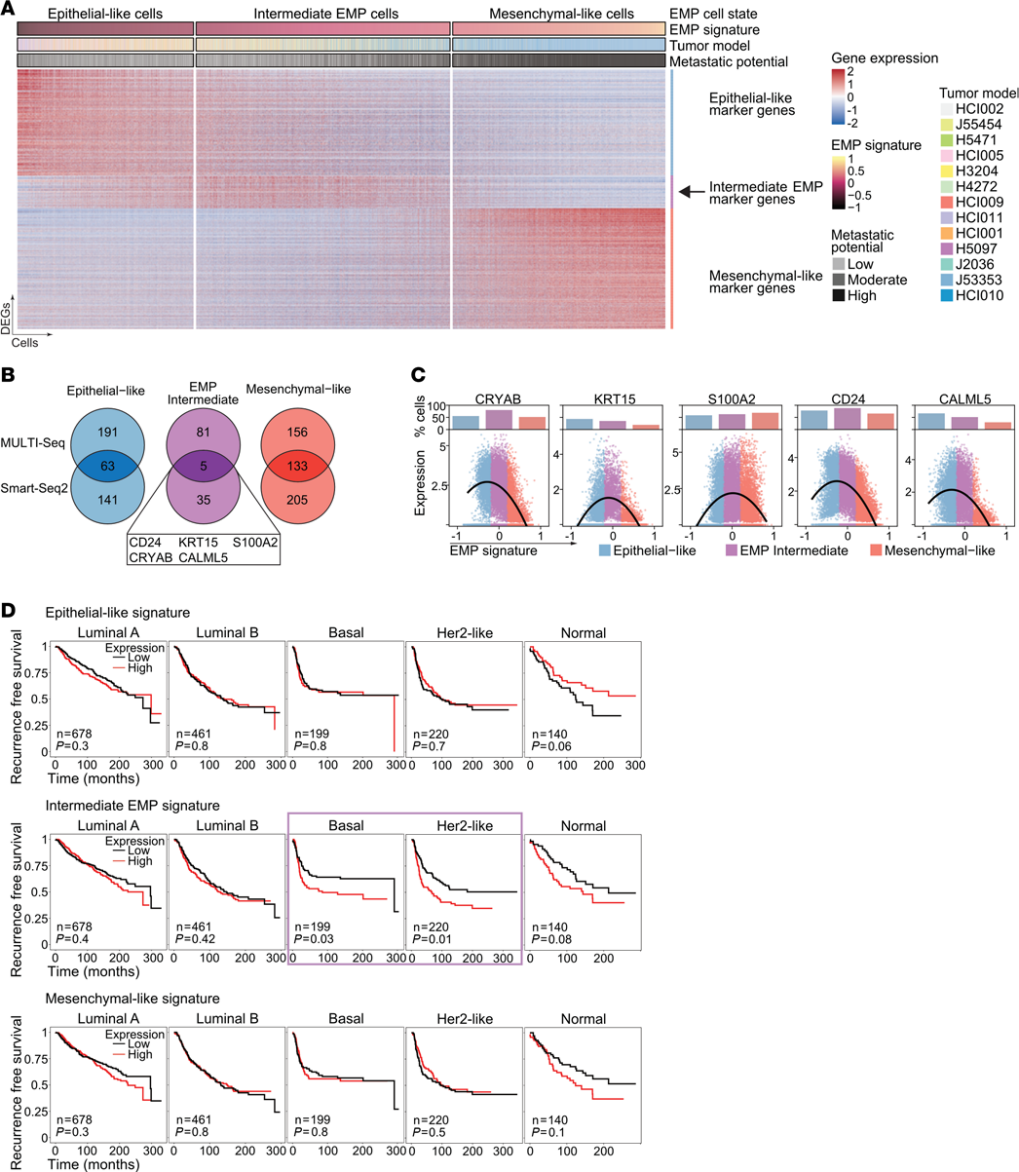
Intermediate EMP cells are characterized by specific markers. (**A**) Heatmap showing expression of DEGs for epithelial-like, mesenchymal-like, and intermediate EMP cells from the MULTI-Seq data. Cells are ordered by increasing EMP signature. Annotations indicate the EMP cell state, EMP signature expression, tumor model, and metastatic potential. The arrow highlights intermediate EMP cell marker genes. (**B**) Venn diagrams showing overlapping DEGs of epithelial-like, mesenchymal-like, and intermediate EMP cells between the Smart-Seq2 and MULTI-Seq data sets. The overlapping markers for intermediate EMP cells are highlighted. (**C**) Scatter plots show expression of the indicated genes in individual cells ordered by increasing EMP signature expression. The dots show expression levels in individual cells, and lines show smoothed expression of expressing cells. The bar charts on top shows the proportion of positively expressing cells for the EMP cell states. The MULTI-Seq data set is shown. (**D**) Kaplan-Meier plots show the RFS of patients with BC (METABRIC) stratified by PAM50 BC subtype using the mean expression of the epithelial-like, intermediate EMP, and mesenchymal-like signatures. The number of patients and *P* value are shown. The purple box indicates data with a significant *P* value calculated by log-rank test.

To further characterize this intermediate EMP cell state, we performed differential gene expression analysis between the 3 EMP cell states in both data sets (Smart-Seq2 and MULTI-Seq) and identified genes upregulated in epithelial-like, intermediate EMP, and mesenchymal-like cells (Figure 5A, Supplemental Figure 6A, and Supplemental Tables 7 and 8). For each EMP cell state, we focused on marker genes that were shared between the 2 data sets (Figure 5B). Surprisingly, only 12% (37 of 303, MULTI-Seq) to 18% (56 of 303, Smart-Seq2) of the published markers (42) that were used to classify the 3 EMP states were among the DEGs. Most of the identified DEGs were exclusive to one or both of our data sets and were not found in the set of published markers (Supplemental Figure 6B). Genes shared across all 3 sets included common mesenchymal (e.g., *VIM*, *BGN*, *SNAI2*, *LOX*) and epithelial (e.g., *KRT18*, *KRT8*) marker genes, whereas other canonical markers, such as *EPCAM*, *CDH1*, *CDH2*, and *FN1*, were not included. Only 5 intermediate EMP cell marker genes were shared between our data sets (Figure 5B). The expression of all 5 intermediate EMP marker genes (*CRYAB*, *KRT15*, *S100A2*, *CD24*, and *CALML5*) peaked in intermediate EMP cells and decreased in epithelial-like and mesenchymal-like cells (Figure 5C and Supplemental Figure 6C).

The EMP marker genes could serve as biomarkers to identify BC patients with an increased proportion of potentially aggressive tumor cells. To test the clinical significance of our findings, we analyzed 2 BC gene expression data sets. In the first data set (41), across different BC subtypes, patients whose tumors showed high expression of the epithelial-like gene signature had better RFS, whereas patients whose tumors showed high expression of the intermediate EMP or mesenchymal-like gene signature had worse RFS (Supplemental Figure 6D). In an independent data set (METABRIC) (46), the intermediate EMP cell gene signature showed a BC subtype–dependent correlation with patient-related outcomes; while there was no correlation in luminal tumors, high expression of the intermediate EMP cell signature was associated with worse RFS in patients with basal or HER2-like BC (Figure 5D). Patients with these subtypes also had poorer outcomes and greater therapeutic resistance than did patients with other subtypes (47).

In summary, we identified intermediate EMP cells that expressed specific marker genes, and high expression of these genes was associated with worse patient-related outcomes. These intermediate EMP cell marker genes could serve as targets for therapies to block the dynamic process of EMP by directly targeting the most potentially plastic cells, thereby interfering with the metastatic cascade.

## Discussion

In this study, we reveal the transcriptional differences between primary BC tumors and their matched lung metastases and how ITH affects metastasis. We characterized a large panel of BC PDX models displaying different metastatic phenotypes. We found significant differences in the transcriptional profiles of metastatic cells compared with their tumors of origin; the transcriptional profiles also showed high patient-to-patient variability. We identified EMP as a shared key feature of ITH across tumor models, revealing a continuum of different EMP cell states. Furthermore, we found that EMT was enriched in primary tumors with a high metastatic potential. Last, we identified markers of intermediate EMP cells and observed that the upregulation of these markers was correlated with patient outcomes.

Differential gene expression analysis revealed strong transcriptional differences between primary tumors and their matched lung metastases. Our analysis suggests that the observed transcriptional differences between primary tumors and metastases were not a result of genomic heterogeneity or clonal selection. However, we cannot completely rule out the possibility that genomic differences exist between individual tumor and metastatic cells, since we used transcriptional data to infer genomic information. Nevertheless, our results align with studies using exome sequencing or lineage tracing, which revealed that lung metastases are polyclonal (48) and that transcriptional differences are not a result of clonal selection (49). We found that primary tumors had consistent upregulation of genes involved in hypoxia, glycolysis, and other metabolic pathways. Metastatic cells showed enrichment of c-MYC signaling and oxidative phosphorylation pathways, which aligns with previous studies investigating BC lung metastases (48, 50, 51). Moreover, metastatic cells frequently showed upregulation of genes involved in cytoskeleton assembly, cell motility, and cellular stress. These transcriptional differences are presumably necessary for the acquisition of traits needed for dissemination and are a result of adaptation to the environment of lung metastasis. Understanding the transcriptional differences between primary tumors and metastatic cells also found in other sites of metastasis could have implications for developing organ-specific therapeutics. Additionally, it will be crucial for future studies to investigate the effect of ITH on the remodeling of nonmalignant cells in the tumor microenvironment, the formation of (pre)-metastatic niches (52), and their consequences for metastasis.

We identified metastatic signatures in the primary tumor cells that are correlated with patient-related outcomes. Other studies investigating human tumor tissue on the individual cell level lack information about patient outcomes, whereas bulk expression data do not appropriately reflect ITH and are often contaminated with nonmalignant cells. Besides these limitations of bulk expression data, they thus far provide the only validation data sets to show clinically relevant expression signatures. Here, we provide a metastatic gene signature solely derived from scRNA-Seq data of tumor cells that are associated with tumor progression and metastasis.

Additionally, we found that markers of EMT were enriched in highly metastatic primary tumors and that EMP was a dominant feature of ITH. We identified epithelial-like, mesenchymal-like, and intermediate EMP cells that surprisingly coexisted in every tumor. Intermediate EMP cells (also previously described as partial-EMT, hybrid-EMT, or EMT cells) have recently been reported to exhibit greater metastatic potential than mesenchymal or epithelial cells in the context of tail-vein injection of skin squamous carcinoma cells or orthotopic injection of highly metastatic pancreatic ductal adenocarcinoma cells, both in genetic mouse models (44, 53). In contrast, we did not observe a correlation between the abundance of intermediate EMP cells in primary tumors and their metastatic potential. Indeed, we found that intermediate EMP cells were also present in poorly metastatic tumor models. Instead, we found that a higher mesenchymal phenotype (high EMP signature) and a higher proportion of mesenchymal-like cells correlated with metastatic potential.

Our findings do not necessarily contradict the observation that intermediate EMP cells are highly metastatic. One explanation may be a cooperativity between different EMP cell states, e.g., a higher proportion of mesenchymal cells may support the metastatic capabilities of intermediate EMP cells. For example, an admixed culture of (epithelial-like) CD24^+^CD44^–^ and (mesenchymal-like) CD24^–^CD44^+^ immortalized normal human mammary epithelial (HMLER) cells was more efficient in mammosphere formation than either cell population alone (54). The observation that (lung) metastases can have polyclonal origins (48, 55) and that circulating tumor cell (CTC) clusters are more effective in metastasis formation than individual CTCs (56) supports our idea that cooperativity between different EMP cell states could result in more effective metastasis formation. Thus, our data suggest the hypothesis that the critical factor that determines the metastatic potential of a tumor is the composition of the entire tumor cell population, i.e., their EMP states and the level of cooperativity among these cells as opposed to the presence or absence of a highly metastatic, potentially small subset of cells.

We found that high expression of intermediate EMP genes was associated with poorer outcomes in a subset of patients with BC, whereas mesenchymal and epithelial gene expression did not show any subtype-specific correlation. This observation not only highlights the potential importance of the intermediate EMP cell state to patient outcomes but also indicates that the EMP process and its involvement in metastatic disease may be subtype specific. Different markers of intermediate EMP cell states have been identified and linked to tumorigenesis, metastasis, and stemness (such as CD104, EPCAM^–^CD106^+^, and ALDH1) (44, 57–59). These studies and ours highlight the need for a deeper understanding of the involvement of the intermediate EMP cell state in metastasis and its potential cell type and spatial-temporal context specificity (7, 21).

Here, we identified 5 marker genes of the intermediate EMP cell state: *CD24*, *CRYAB*, *KRT15*, *CALML5*, and *S100A2*. These markers have been previously implicated in EMT, cancer stemness, and metastasis pathways. For example, the cell-surface protein CD24 shows contradictory results regarding its role in tumor progression. Whereas CD24^–/lo^CD44^+^ cells have been shown to initiate breast tumors in NOD/SCID mice (60), high *CD24* expression increases metastasis (61), and CD24^+^CD90^+^ cells initiate metastases that display a mesenchymal phenotype (62, 63). Another identified intermediate EMP marker, *CRYAB*, encodes the small heat shock protein α–basic–crystallin (αB-crystallin), confers anoikis resistance, and thereby enables metastatic dissemination (64). *CRYAB* is overexpressed in various tumors (65, 66), including the basal BC subtype (67), and has been associated with poor patient outcomes and metastasis (68–71). Importantly, *CRYAB* is highly expressed in a small, nonproliferative or dormant metastatic cell population and might be required for the survival of single metastatic cells and micrometastasis in the brain and lung (72, 73). Moreover, *KRT15*, *CALML5*, and *S100A2* have been described as having stem cell functions and EMP regulation, suggesting both tumor-promoting and tumor-suppressive roles (74–78). Collectively, these data suggest that our identified intermediate EMP cell markers could indeed mark a plastic intermediate EMP cell state with potential stem-like properties and that cooperativity may exist between cell populations with different EMP characteristics. Future work may use these makers to investigate the metastatic function of intermediate EMP cells in greater detail and develop targeted therapies to inhibit metastasis.

## Methods

### Sex as a biological variable.

In this study, we exclusively used female mice for the animal studies and publicly available data from BC that primarily affects women. We cannot draw any conclusion if our findings apply to male BC that comprise approximately less than 1% of all BC cases (79). We cannot draw any conclusion if our findings apply to male BC that compromise approximately less than 1% of all BC cases.

### PDX experiments.

Fresh primary breast tumor tissues were cut into 1 mm thick pieces and orthotopically transplanted into cleared mammary fat pads of 4-week-old NOD/SCIDγ mice (The Jackson Laboratory) to generate novel PDX models (J53353, J2036, and J55454, Supplemental Table 1). Established PDX lines were provided by M.T. Lewis (Baylor College of Medicine, Houston, Texas, USA) and A. Welm (University of Utah, Huntsman Cancer Institute, Salt Lake City, Utah, USA) and transplanted as previously described (24, 27, 80). Once palpable, tumors were measured 2 times per week using a caliper to monitor growth kinetics. Tumor volume was calculated using the following formula:
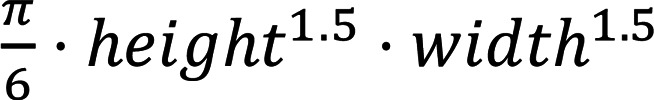


Unless otherwise noted, all PDX animals were euthanized at the endpoint, when the primary tumor reached 2.5 cm in diameter. In resection experiments involving HCI002, tumors were surgically removed at 1.0–2.0 cm in diameter. Metastases were allowed to grow in animals that underwent resection until the endpoint was reached (including a 2.5 cm diameter of the recurrent tumor). At the endpoint, the primary tumor and metastatic lungs were harvested, cut into small pieces, and cryopreserved using Recovery Cell Culture Freezing Medium (Thermo Fisher Scientific, 12648010) and stored in liquid nitrogen until further analysis.

### Histology and tissue staining.

For each PDX animal, after dissection, the middle and postcaval lobes of the right lung were fixed in 4% paraformaldehyde overnight and processed for paraffin embedment. For immunohistochemistry, tissues were stained using the following antibodies: ER (Abcam, ab1660, 1:200) using Ventana Discovery Ultra Automated Slide Stainer and Cell Conditioning 1 and DAB, PR (Cell Signaling Technology, 8757S, 1:1,000); ER (Cell Atlas,HPA000450; 1:1,000) and HER2 (Cell Atlas, HPA001383, 1:200) were manually stained using AEC Chromogen (Sigma-Aldrich, AEC101) as a substrate following standard protocols (81). For histological analysis, tissue sections were stained with H&E using standard protocols. Tissue slides were scanned (Zeiss Axio ScanZ.1, 3DHistech Pannoramic SCAN II Scanner), and images were analyzed using QuPath. Metastatic foci were easily identified by a larger nuclei/cytoplasm ratio. Micrometastases were defined as fewer than 10 tumor cells, intermediate metastatic foci as 10–100 cells, and macrometastases as more than 100 cells. The number and total area of metastatic foci and the total tissue area were determined.

### ScRNA-Seq experiments, library preparation, and data analysis.

Detailed information regarding scRNA-Seq library preparation and data analysis can be found in the Supplemental Methods.

Additional details on methods can be found in the Supplemental Methods.

### Statistics.

Full *P* values are reported, and *P* values of less than 0.05 were viewed as significant. Survival was estimated using the Kaplan-Meier method and analyzed by log-rank test. Comparisons between 2 groups were tested using a 2-tailed, unpaired Wilcoxon rank or Fisher’s exact test. The median or mean is shown as indicated in the figure legends. The lower and upper hinges correspond to the first and third quartiles (the 25th and 75th percentiles).

### Study approval.

Fresh primary breast tumor samples were obtained from the Cooperative Human Tissue Network (CHTN) with the approval of the IRB of UCSF. Tissues were received as deidentified samples, and all donors provided written informed consent. Medical reports were obtained without personally identifiable information. The UCSF IACUC reviewed and approved all animal experiments.

### Data availability.

Raw sequencing files are available under the NCBI BioProject number PRJNA847563. Raw and processed data have been deposited in the NCBI’s Gene Expression Omnibus (GEO) database (GEO GSE210283). Processed data are available as h5ad files on figshare (https://figshare.com/s/328942c0b8dc9aa69be1 and https://figshare.com/s/b53f327a8b612a7b2eeb). Code is available on GitHub (https://github.com/aopisco/scBC; Commit ID: e8c19728b483efcb6b08cb90a2dd00bced727d04; Commit URL: https://github.com/aopisco/scBC/commit/e8c19728b483efcb6b08cb90a2dd00bced727d04). A Supporting Data Values file is provided in the supplemental materials.

## Author contributions

JW conceptualized the study. WT, JW, CSM, MYH, DS, and AA analyzed data. JW, CMMD, SH, AN, VS, and EA performed animal studies. JW, AA, JHH, WT, CMMD, and JVL performed tissue processing. JW and CSM prepared MULTI-Seq libraries. WT, MB, and JW prepared Smart-Seq2 libraries. AMD and NN performed sequencing. JW, CMMD, AA, LA, SH, PSW, and NS performed tissue staining. JW, S Durney, and SYL performed tissue imaging and analysis. JW and WT wrote the manuscript. S Darmanis, AG, MYH, AOP, and CSM edited the manuscript. S Darmanis, AOP, AG, ZJG, and ZW provided guidance and funding. HSR provided funding.

## Supplementary Material

Supplemental data

Supplemental table 1

Supplemental table 2

Supplemental table 3

Supplemental table 4

Supplemental table 5

Supplemental table 6

Supplemental table 7

Supplemental table 8

Supporting data values

## Version 1 09/03/2024

Electronic publication
