# Supplementary material for: Single-cell analysis of breast cancer metastasis reveals epithelial-mesenchymal plasticity signatures associated with poor outcomes
Source: J Clin Invest. 2024 Sep 3;134(17):e164227. doi: 10.1172/JCI164227 (PMC11364385; doi:10.1172/JCI164227)
Supplement: Supplemental data [file jci-134-164227-s194.pdf]

Supplementary Information

Methods

Sex as a biological variable

In this study, we used exclusively female mice for animal studies and publicly available data from BC that majorly affects women. We can draw any conclusion if our findings apply to male BC that compromise about <1% of all BC cases (1).

PDX experiments

Fresh primary breast tumor tissues were cut into 1-mm-thick pieces and orthotopically transplanted into cleared mammary fat pads of 4-week-old NOD-SCID gamma mice to generate novel PDX models (J53353, J2036, and J55454, Supplementary Table 1). Established PDX lines were kindly provided by M.T. Lewis (Baylor College of Medicine, Houston, USA) and A. Welm (University of Utah, Huntsman Cancer Institute, Salt Lake City, USA) and transplanted in the same way and as previously described (2–4). Once palpable, tumors were measured 2x/week using a caliper to monitor growth kinetics. Tumor volume was calculated using the following formula:

$$\frac{\pi}{6} \cdot height^{1.5} \cdot width^{1.5}.$$

Unless otherwise noted, all PDX animals were euthanized at the endpoint, when the primary tumor reached 2.5 cm in diameter. In resection experiments involving HCI002, tumors were surgically removed at 1.0–2.0 cm in diameter. Metastases were allowed to grow in animals that underwent resection until the endpoint was reached (including 2.5 cm diameter of recurrent tumor). At the endpoint, the primary tumor and metastatic lungs were harvested, cut into small pieces and cryopreserved using Recovery Cell Culture Freezing Medium (Thermo Fisher #12648010) and stored in liquid nitrogen until further analysis.

Histology and tissue staining

For each PDX animal, after dissection, the middle and postcaval lobes of the right lung were fixed in 4% PFA overnight and processed for paraffin embedding. For immunohistochemistry, tissues were stained using the following antibodies: ER (abcam, ab1660, 1:200) using Ventana Discovery Ultra automated slide stainer and cell conditioning 1 and DAB, PR (Cell Signaling, 8757S, 1:1000), ER (Cell Atlas; HPA000450; 1:1000) and HER2 (Cell Atlas, HPA001383; 1:200) were manually stained using AEC Chromogen (Sigma-Aldrich, AEC101) as a substrate following standard protocols (5). For histological analysis, tissue sections were stained with hematoxylin and eosin using standard protocols. Tissue slides were scanned (Zeiss Axio ScanZ.1, 3DHistech Pannoramic SCAN II Scanner) and images were analyzed using QuPath. Metastatic foci were easily identified by a larger nuclei/cytoplasm ratio. Micrometastases were defined as < 10 tumor cells, intermediate metastatic foci as 10-100 cells and macrometastases as >100 cells. The number and total area of metastatic foci and the total tissue area were determined.

Lysis plate preparation

Lysis plates were prepared by dispensing 0.4 µL lysis buffer (0.5 U Recombinant RNase Inhibitor (Takara Bio, 2313B), 0.0625% Triton™ X-100 (Sigma, 93443-100ML), 3.125 mM dNTP mix (Thermo Fisher, R0193), 3.125 µM oligo-dT_{30}VN (IDT, 5′AAGCAGTGGTATCAACGCAGAGTACT_{30}VN-3′) and 1:600,000 ERCC RNA spike-in mix (Thermo Fisher, 4456740)) into 384-well hard-shell PCR plates (Bio-Rad HSP3901) using a Dragonfly liquid handler (STP Labtech). All plates were then spun down for 1 minute at 3220xg and snap-frozen on dry ice. Plates were stored at −80°C until FACS.

Sample preparation and FACS sorting

Primary tumor and metastatic lung tissues derived from the different PDX models were processed, stained, MULTI-seq labeled and FACS sorted as previously described (6). In brief,

tissues were thawed, and dissociated in digestion media containing 50 µg/ml Liberase TL (Sigma-Aldrich) and 2 × 104 U/ml DNase I (Sigma-Aldrich) in DMEM/F12 (Gibco) using standard GentleMacs (37C_m_LDK_1, 37_m_TDK1) protocols. Washed and filtered single-cell suspensions were stained with viability dye (Zombie NIR, 1:500, BioLegend, no. 423105), blocked with Fc-block (1:200, Tonbo, 70-0161-U500), and with LIN (anti-mouse TER119-FITC, Thermo Fisher, 11-5921-82; anti-mouse CD31-FITC, Thermo Fisher, 11-0311-85; anti-mouse CD45-BV450, Tonbo, 75-0451-U100; anti-mouse MHC-I-APC, eBioscience, 17-5999-82) and anti-human CD298 (PE, BioLegend, 341704).

For the Smart-Seq2 experiments, tissues derived from the different PDX models were processed as described above, and primary tumor and metastatic cells were sorted directly into cooled lysis plates and snap-frozen for library preparation. If multiple plates were sorted from one PDX model, each plate contained half primary tumor and half metastatic cells to avoid plate-specific batch effects. For the MULTI-seq experiments, MULTI-seq LMO barcode anchors and coanchors were used at a final concentration of 2.5 µM directly after antibody staining before FACS sorting as described previously(6). For one experiment (PDX1), we used sets of three unique MULTI-seq barcodes/sample. After sorting, enriched live, LIN^-/$hCD298^+$ cells were pooled and loaded into 10x microfluidics lanes at an average loading concentration of approximately 30,000 cells/lane.

cDNA synthesis and library preparation

cDNA synthesis was performed using the Smart-seq2 protocol (7–9). Briefly, 384-well plates containing single-cell lysates were thawed on ice, followed by first-strand synthesis. Then, 0.6 µL of reaction mix (16.7 U/µl SMARTScribe Reverse Transcriptase (Takara Bio, 639538), 1.67 U/µl Recombinant RNase Inhibitor (Takara Bio, 2313B), 1.67X First-Strand Buffer (Takara Bio, 639538), 1.67 µM TSO (Exiqon, 5′-AAGCAGTGGTATCAACGCAGACTACATrGrG+G-3′), 8.33 mM DTT (Bioworld, 40420001-1), 1.67 M betaine (Sigma, B0300-5VL), and 10 mM $MgCl_2$ (Sigma, M1028-10X1ML)) was added to each well using a Dragonfly liquid handler (STP Labtech).

Reverse transcription was carried out by incubating the plates on a ProFlex 2x384 thermal-cycler (Thermo Fisher) at 42°C for 90 min and stopped by heating at 70°C for 5 min.

Subsequently, 1.5 µL of PCR mix (1.67X KAPA HiFi HotStart ReadyMix (Kapa Biosystems, KK2602), 0.17 µM IS PCR primer (IDT, 5′-AAGCAGTGGTATCAACGCAGAGT-3′), and 0.038 U/µl lambda exonuclease (NEB, M0262L)) was added to each well with a Dragonfly liquid handler (STP Labtech), and second strand synthesis was performed on a ProFlex 2x384 thermal cycler by using the following program: 1. 37°C for 30 minutes, 2. 95°C for 3 minutes, 3. 23 cycles of 98°C for 20 s, 67°C for 15 s, and 72°C for 4 minutes, and 4. 72°C for 5 minutes.

The amplified product was diluted 1:10 with 10 mM Tris-HCl (Thermo Fisher, 15568025). Then, 0.6 µL of diluted product was transferred to a new 384-well plate using the Viaflow 384 channel pipette (Integra). Illumina sequencing libraries were prepared using a library preparation protocol modified from previously reported tagmentation-based protocols (9, 10). Briefly, tagmentation was carried out by mixing each well with 1 µL of 1.6x Homebrew Tn5 Tagmentation Buffer and 0.2 µL of Homebrew Tn5 enzyme, and then incubated at 55°C for 3 min. The reaction was stopped by adding 0.4 µL of 0.1% sodium dodecyl sulfate (Fisher Scientific, BP166-500) and centrifuging at room temperature at 3,220 g for 5 min. Indexing PCRs were performed by adding 0.4 µL of 5 µM i5 indexing primer, 0.4 µL of 5 µM i7 indexing primer, and 1.2 µL of Nextera NPM mix (Illumina). All reagents were dispensed with Mosquito liquid handlers (STP Labtech). PCR amplification was carried out on a ProFlex 2x384 thermal cycler using the following program: 1. 72°C for 3 minutes, 2. 95°C for 30 s, 3. 12 cycles of 98°C for 10 s, 67°C for 30 s, and 72°C for 1 minute, and 4. 72°C for 5 minutes.

Library sequencing

Following library preparation, the wells of each library plate were pooled using a Mosquito liquid handler (STP Labtech). The pooling was followed by two rounds of purifications using 0.7x AMPure beads (Fisher, A63881). Library quality was assessed using high-sensitivity capillary

electrophoresis on a TapeStation (Agilent), and libraries were quantified by qPCR (Kapa Biosystems, KK4923) on a CFX96 Touch Real-Time PCR Detection System (Bio-Rad). Plate pools were normalized to 2 nM and equal volumes from the library plates were mixed to generate the sequencing sample pool.

Sequencing libraries from 384-well plates

Libraries were sequenced on the NextSeq or NovaSeq 6000 Sequencing System (Illumina) using 2 × 100 bp paired-end reads and 2 × 12 bp index reads. NextSeq runs used high output kits, whereas NovaSeq runs used a 300-cycle kit (Illumina, 20012860). The PhiX control library was spiked in at ~1%.

Sequencing libraries for MULTI-seq

For the MULTI-seq dataset, gene expression library preparation was performed using the v2 10x library kit with modifications as described previously to generate MULTI-seq libraries(6).

Data analysis

Data extraction

For Smart-Seq2, sequences from the NovaSeq or NextSeq were demultiplexed using bcl2fastq v.2.19.0.316. Reads were aligned to the GENCODE V30 genome using STAR v.2.5.2b with parameters TK. Gene counts were calculated using HTSEQ v.0.6.1p1 with default parameters, except that ‘stranded’ was set to ‘false’, and ‘mode’ was set to ‘intersection-nonempty’. For MULTI-seq, sequences from the microfluidic droplet platform were demultiplexed and aligned using CellRanger v.5.0.1, available from 10x Genomics with default parameters.

MULTI-seq demultiplexing

MULTI-seq barcode FASTQs were converted to barcode unique molecular modifier (UMI) count matrices using the ‘MULTIseq.preProcess’ and ‘MULTIseq.align’ functions in the deMULTIplex R

package (6) with default parameters. Notably, 'PDX3' FASTQs were randomly downsampled to $1x10^8$ total reads before UMI count matrix conversion to minimize computation time. Next, since cells labeled with the same MULTI-seq barcodes were split across multiple 10x Genomics microfluidics lanes in each experiment, MULTI-seq UMI count matrices from each lane were concatenated (note for clarity: PDX1 and PDX3 matrices were concatenated separately) to maximize classification performance. Using these concatenated matrices, samples were then classified into sample groups using the deMULTIplex workflow described previously (with semisupervised negative-cell reclassification) (6). Notably, since samples in the PDX1 experiment were encoded by sets of three unique MULTI-seq barcodes, classification was performed on the median cell barcode count for each set. Moreover, cells with the top and bottom 5% of MULTI-seq barcode counts were masked during the initial classification workflow in the PDX1 dataset, and were reintroduced as 'negatives' during semisupervised negative cell reclassification. Analogous barcode count merging and outlier masking were not necessary for the PDX3 data, which were classified successfully using the default deMULTIplex workflow.

Data preprocessing

For Smart-seq 2 data, gene count tables were combined with the metadata variables using the Scanpy Python package version 1.8.1 (11). We removed the genes that were not expressed in at least 5 cells. Cells with counts less than 5000 or fewer than 500 detected genes were removed. Additionally, we removed cells with more than 50% mitochondrial genes and 20% ERCC reads. The data were then normalized using size factor normalization (such that every cell had gene counts of 10,000) and then log-transformed. We selected the top 2000 genes with the highest standardized variance as the highly variable genes by using the VST method in Seurat V3 (12), which is also implemented in Scanpy. Cell-cycle regression was performed after calculating the score of the S and G2/M phases for each cell (13). The data were then scaled to a maximum value of 10. We then computed principal component (PC) analysis and neighborhood graphs and

clustered the data using Louvain and Leiden methods. The data were visualized using UMAP projection.

For MULTI-seq data, for each 10X lane, we first removed the cells with UMI counts below 2500, and fewer than 250 genes, or more than 50% mitochondrial reads by using the Scanpy Python package version 1.8.1. In addition, to filter out reads from ambient RNA, we ran DecontX(14) with default parameters separately for each 10X lane. Next, we refiltered the dataset from every 10X run when cells did not contain a minimum number of genes (250), minimum of counts/UMIs (2500), and/or had more than 50% mitochondrial reads. The data were then further processed as described above for the Smart-Seq2 dataset. Cells were sample assigned using the MULTI-seq demultiplexing result, thereby removing doublets but including unassigned ‘negative’ cells. We used DBSCAN clustering to recover MULTI-seq-unassigned ‘negative’ cells. Based on the results of the Smart-Seq2 data, cells from different PDX tumors clustered separately from each other in transcriptional space. Negative cells in one DBSCAN cluster were assigned as the same tumor sample as the majority of MULTI-seq classified cells in that cluster. After completing cell assignment, all 10X runs were combined into one MULTI-seq dataset, and genes that were not expressed in at least 20 cells were removed. We then performed normalization, log-transformation, identification of highly variable genes, cell cycle regression, principal component analysis, UMAP dimension reduction, and Louvain and Leiden clustering as described for the Smart-Seq data.

EMP scoring and classification

We used gene set enrichment analysis (GSVA) scoring with its default parameters to assign each cell an epithelial (E-score) and mesenchymal score (M-score) using epithelial and mesenchymal marker genes (15). The EMP-score for each cell is calculated by the sum of the E-score and M-score for that cell. Cells with an EMP-score > 0.2 were classified as mesenchymal-like cells, cells

with an EMP-score < -0.2 were classified as epithelial-like cells, and cells with an EMP-score between -0.2 and 0.2 were classified as EMP intermediate cells.

Cell phase proportion statistical test

Cells were assigned to different cell cycle phases based on the cell cycle score calculated previously. Then cell phase proportions in each tumor were calculated in different groups in each category, such as EMP cell stage, sort, and metastatic potential group. Finally, the Wilcoxon rank test was performed to compare groups in each category in each cell phase. The statistical tests were generated by using the Seaborn (16) and Statannot (17) packages in Python.

ROC curve and AUC value

The cells were first ordered by their PC2 value either in the whole SS2 dataset or in individual tumor samples. The true and false-positive rates were calculated based on the cell's label (primary tumor cell or metastatic cell) and PC2 value by using the "roc_curve" function from Scikit-learn (18). In addition, "roc_auc_score" from Scikit-learn was used to calculate the AUC value from the true- and false-positive rates.

Differentially expressed genes

Identifying DEGs in primary tumor and metastatic cells

We performed differential expression analysis between primary tumor and metastatic cells in the entire dataset using the Seurat function FindMarkers using MAST (19) and the tumor model as the latent variable. In addition, we identified DEGs between primary tumor and metastatic cells for each tumor sample separately using the same Seurat function without setting the latent variable. Genes with p value < 0.05 and log2-fold change > 0.5 were retained for further analysis. After filtering, we combined the DEGs from tumors within the same metastatic potential group. We included DEGs that are shared between at least two tumors in the same metastatic potential group.

Identifying gene signatures associated with metastatic potential

To identify genes in the primary tumor that are associated with metastatic potential we first removed metastatic cells from the data. Then, we used the Seurat function FindMarkers using the MAST test and tumor model as the latent variable for identifying DEGs between one individual tumor and all tumors in the other metastatic groups. Genes were filtered with a cutoff of p value < 0.05 and log2-fold change > 0.5. After filtering, we combined the upregulated gene lists from tumors within the same metastatic potential group. Signature genes related to metastatic potential were determined as genes that were shared between at least two tumors in the same metastatic potential group. As final signature genes, we selected genes that were shared between the SS2 and MULIT-Seq datasets. The number of overlapping DEGs between those two scRNASeq methods was shown to vary between tissues and cell types and was within the expected range (8, 20).

Identifying EMP marker genes

We identified marker genes for each EMP category (epithelial-like, EMP intermediate, mesenchymal-like) using the Seurat function FindMarkers using the MAST test and tumor sample ID as the latent variable. Genes were filtered with a cutoff of p values < 0.05 and log2-fold change > 0.5.

Gene set enrichment analysis

To identify pathways that were enriched in primary tumor or metastatic cells, we used the fgsea package(21) with Hallmark and GO gene sets from MSigDB (22, 23). We examined pathways that were significantly enriched in at least four tumor models. Pathways enriched in highly and poorly metastatic signatures were identified with the online tool of MSigDB.

Copy number variation analysis

To investigate clonal relationships between individual cells derived from primary tumors and metastases we inferred copy number variation (CNV) profiles for each cell using the inferCNV package(24) with the SS2 data set of mammary epithelial cells as a reference (20) and using the denoised output generated with default parameters. We calculated the mean CNV values for each genome position across all cells derived from primary tumors and metastases for each tumor and determined the Pearson correlations for each tumor model.

Survival analysis

For survival analysis, we used the breast cancer gene chip mRNA dataset from the KM-plotter (25) website. The mean expression levels of the signature genes were calculated for each sample in the dataset. The patient samples were then divided into low- and high-expressing sample groups with the median expression as the cutoff. Visualization was performed using the Lifelines Python package (26). The metastatic potential gene lists resulted from the overlapping genes between the MULTI-seq and Smart-Seq2 datasets from each metastatic potential group. For EMP signature gene lists, the epithelial signature gene list and mesenchymal signature gene list were the overlapping genes from the top 100 differentially expressed genes in the MULTI-seq and Smart-Seq2 datasets, and the intermediate EMP marker gene signature included the overlapping genes found in both the MULTI-seq and Smart-Seq2 datasets.

In addition, the METABRIC(27) dataset was obtained from cBioPortal. We used "all_sample_Zscores" to create Kaplan-Meier survival plots (26) and perform log-rank tests for each breast cancer subtype using the mean expression of the intermediate EMP marker genes.

Data Availability

Raw sequencing files are available at the NCBI BioProject number PRJNA847563. Raw and processed data have been deposited in NCBI's Gene Expression Omnibus and are accessible

through GEO Series accession number GSE210283. Processed data are available as h5ad files on figshare (https://figshare.com/s/328942c0b8dc9aa69be1 and https://figshare.com/s/b53f327a8b612a7b2eeb). Code is available on GitHub https://github.com/aopisco/scBC. Supporting data values file is provided as supplementary information.

Statistics

The used statistical test is indicated in the figure legends. Full p values are reported and p values < 0.05 were viewed as significant. Survival was estimated using the Kaplan-Meier method and analyzed by log-rank test. Comparisons between two groups were tested using two-tailed unpaired Wilcoxon rank or Fisher's exact test.

Study approval

Fresh primary breast tumor samples were obtained from the Cooperative Human Tissue Network (CHTN) in accordance with the Institutional Review Boards' approval. Tissues were received as deidentified samples and all subjects provided written informed consent. Medical reports were obtained without personally identifiable information. The UCSF Institutional Animal Care and Use Committee (IACUC) reviewed and approved all animal experiments.

References

1. Siegel RL, Miller KD, Jemal A. Cancer statistics, 2019. *CA Cancer J Clin*. 2019;69(1):7–34.

2. Derose YS, et al. Tumor grafts derived from women with breast cancer authentically reflect tumor pathology , growth , metastasis and disease outcomes. *Nat Med*. 2011;17(11):1514–1520.

3. Zhang X, et al. A Renewable Tissue Resource of Phenotypically Stable, Biologically and Ethnically Diverse, Patient-Derived Human Breast Cancer Xenograft Models. *Cancer Res*. 2013;73(15):4885–4897.

4. Lawson DA, et al. The cleared mammary fat pad transplantation assay for mammary epithelial organogenesis. *Cold Spring Harb Protoc*. 2015;2015(12):1064–1068.

5. Kim S-W, Roh J, Park C-S. Immunohistochemistry for Pathologists: Protocols, Pitfalls, and Tips. *J Pathol Transl Med*. 2016;50(6):411–418.

6. McGinnis CS, et al. MULTI-seq: sample multiplexing for single-cell RNA sequencing using lipid-tagged indices. *Nat Methods*. 2019;16(7):619–626.

7. Picelli S, et al. Full-length RNA-seq from single cells using Smart-seq2. *Nat Protoc*. 2014;9:171.

8. Tabula T, Consortium M. Single-cell transcriptomics of 20 mouse organs creates a Tabula Muris. *Nature*. 2018;562(7727):367–372.

9. Picelli S, et al. Tn5 transposase and tagmentation procedures for massively scaled sequencing projects. *Genome Res*. 2014;24(12):2033–2040.

10. Hennig BP, et al. Large-Scale Low-Cost NGS Library Preparation Using a Robust Tn5 Purification and Tagmentation Protocol. *G3 Bethesda Md*. 2018;8(1):79–89.

11. Wolf FA, Angerer P, Theis FJ. SCANPY: large-scale single-cell gene expression data analysis. *Genome Biol*. 2018;19(1):15.

12. Stuart T, et al. Comprehensive Integration of Single-Cell Data. *Cell*. 2019;177(7):1888-1902.e21.

13. Kowalczyk MS, et al. Single-cell RNA-seq reveals changes in cell cycle and differentiation programs upon aging of hematopoietic stem cells. *Genome Res*. 2015;25(12):1860–1872.

14. Yang S, et al. Decontamination of ambient RNA in single-cell RNA-seq with DecontX. *Genome Biol*. 2020;21(1):57.

15. Tan TZ, et al. Epithelial-mesenchymal transition spectrum quantification and its efficacy in deciphering survival and drug responses of cancer patients. *EMBO Mol Med*. 2014;6(10):1279–93.

16. Waskom ML. seaborn: statistical data visualization. *J Open Source Softw*. 2021;6(60):3021.

17. Florian Charlier, Marc Weber, Dariusz Izak, Emerson Harkin, Marcin Magnus, Joseph Lalli, Louison Fresnais, Matt Chan, Nikolay Markov, Oren Amsalem,, Sebastian Proost, Agamemnon Krasoulis, getzze, & Stefan Repplinger. Statannotations (v0.6). *Zenodo*. [published online ahead of print: 2022]. https://doi.org/10.5281/zenodo.7213391.

18. Pedregosa F, et al. Scikit-Learn: Machine Learning in Python. *J Mach Learn Res*. 2011;12(null):2825–2830.

19. Finak G, et al. MAST: a flexible statistical framework for assessing transcriptional changes and characterizing heterogeneity in single-cell RNA sequencing data. *Genome Biol*. 2015;16(1):278.

20. Jones RC, et al. The Tabula Sapiens: A multiple-organ, single-cell transcriptomic atlas of humans. *Science*. 2022;376(6594). https://doi.org/10.1126/science.abl4896.

21. Gennady Korotkevich, et al. Fast gene set enrichment analysis. *bioRxiv*. 2021;060012.

22. Subramanian A, et al. Gene set enrichment analysis: a knowledge-based approach for interpreting genome-wide expression profiles. *Proc Natl Acad Sci U S A*. 2005;102(43):15545–15550.

23. Liberzon A, et al. The Molecular Signatures Database Hallmark Gene Set Collection. *Cell Syst*. 2015;1(6):417–425.

24. inferCNV of the Trinity CTAT Project. https://github.com/broadinstitute/inferCNV. Accessed January 5, 2024.

25. Győrffy B. Survival analysis across the entire transcriptome identifies biomarkers with the highest prognostic power in breast cancer. *Comput Struct Biotechnol J*. 2021;19:4101–4109.

26. Cameron D-P. lifelines: survival analysis in Python. *J Open Source Softw*. 2019;4(40):1317.

27. Curtis C, et al. The genomic and transcriptomic architecture of 2,000 breast tumours reveals novel subgroups. *Nature*. 2012;486(7403):346–52.

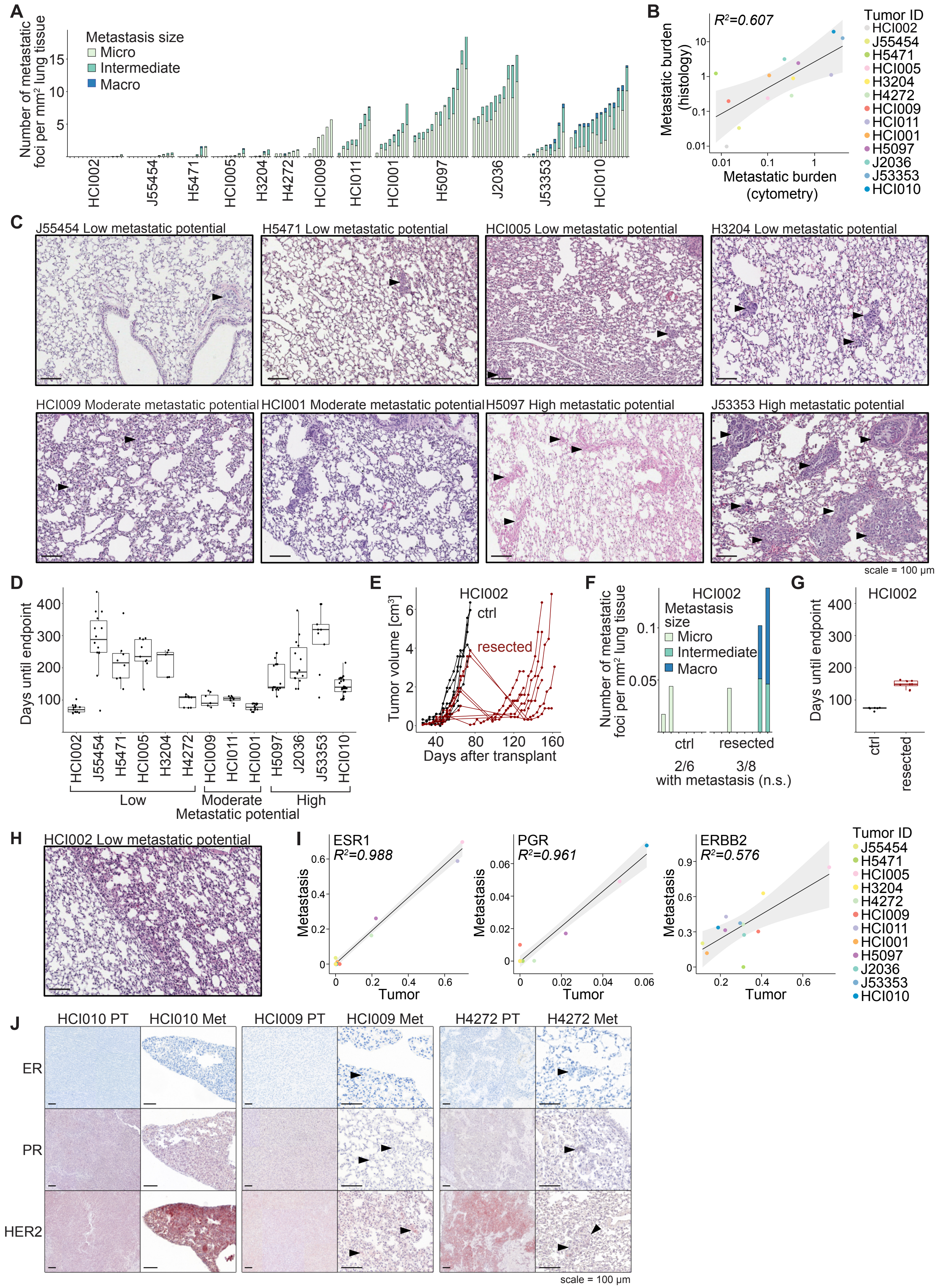
A
Metastasis size
Micro
Intermediate
Macro
Number of metastatic foci per mm² lung tissue
HCI002
J55454
H5471
HCI005
H3204
H4272
HCI009
HCI011
HCI001
H5097
J2036
J53353
HCI010
B
R²=0.607
Metastatic burden (histology)
Metastatic burden (cytometry)
Tumor ID
C
J55454 Low metastatic potential
H5471 Low metastatic potential
HCI005 Low metastatic potential
H3204 Low metastatic potential
HCI009 Moderate metastatic potential
HCI001 Moderate metastatic potential
H5097 High metastatic potential
J53353 High metastatic potential
scale = 100 µm
D
Days until endpoint
Low
Moderate
High
Metastatic potential
E
HCI002
ctrl
resected
Tumor volume [cm³]
Days after transplant
F
HCI002
Metastasis size
Micro
Intermediate
Macro
Number of metastatic foci per mm² lung tissue
ctrl
resected
2/6
3/8
with metastasis (n.s.)
G
HCI002
Days until endpoint
ctrl
resected
H
HCI002 Low metastatic potential
I
ESR1
R²=0.988
PGR
R²=0.961
ERBB2
R²=0.576
Metastasis
Tumor
Tumor ID
J
HCI010 PT
HCI010 Met
HCI009 PT
HCI009 Met
H4272 PT
H4272 Met
ER
PR
HER2
scale = 100 µm

Supplementary Figure 1: Biological characteristics of the PDX models

A) Bar chart showing the number of metastatic foci per mm^2 lung tissue area for individual animals, ordered by increasing metastatic potential of the tumor models. Each bar represents one animal. Metastatic foci are classified as micrometastasis (< 10 cells), intermediate (10-100 cells), and macrometastasis (> 100 cells).

B) Scatter plot showing the correlation of mean metastatic burden as assessed by histology (proportion of metastatic to total lung tissue area) and flow cytometry (proportion of metastatic cells to number of live cells) in each model. Linear regression with 95% confidence intervals and Pearson correlation coefficients are shown.

C) Representative H&E images of metastatic lung tissue for low, moderate and high metastatic potential models. Scale = 100 µm.

D) Boxplot showing median number of days after tumor transplantation until endpoint per model ordered by the metastatic potential as determined in Fig.1B.

E) Spider plot showing tumor growth of HCI002 transplantation alone (black) or transplantation and resection with subsequent recurrence (red).

F) Bar chart showing the number of metastatic foci per mm^2 lung tissue area for HCI002 and HCI002 resected animals. Metastatic foci are classified as described in A. Each tick mark represents one animal. 2/6 (control) and 3/8 animals (resected) developed metastases (p value=1, Fisher's exact test).

G) Boxplot showing the median number of days of tumor transplantation until endpoint comparing HCI002 (black) and HCI002 resection (red).

H) Representative H&E images of lung tissue of HCI002 demonstrating low metastatic potential.

I) Scatterplots showing the correlation of the mean expression of the indicated receptors in primary tumor and metastatic cells per model. Linear regressions with 95% confidence intervals and Pearson correlation coefficients are shown.

J) Representative images showing immunohistochemistry staining for ER, PR, HER2 for primary tumor (PT) and metastatic lung (Met) for TNBC models. Arrows indicate metastasis. When possible, the same metastasis is shown in consecutive sections. Scale = 100 µm.

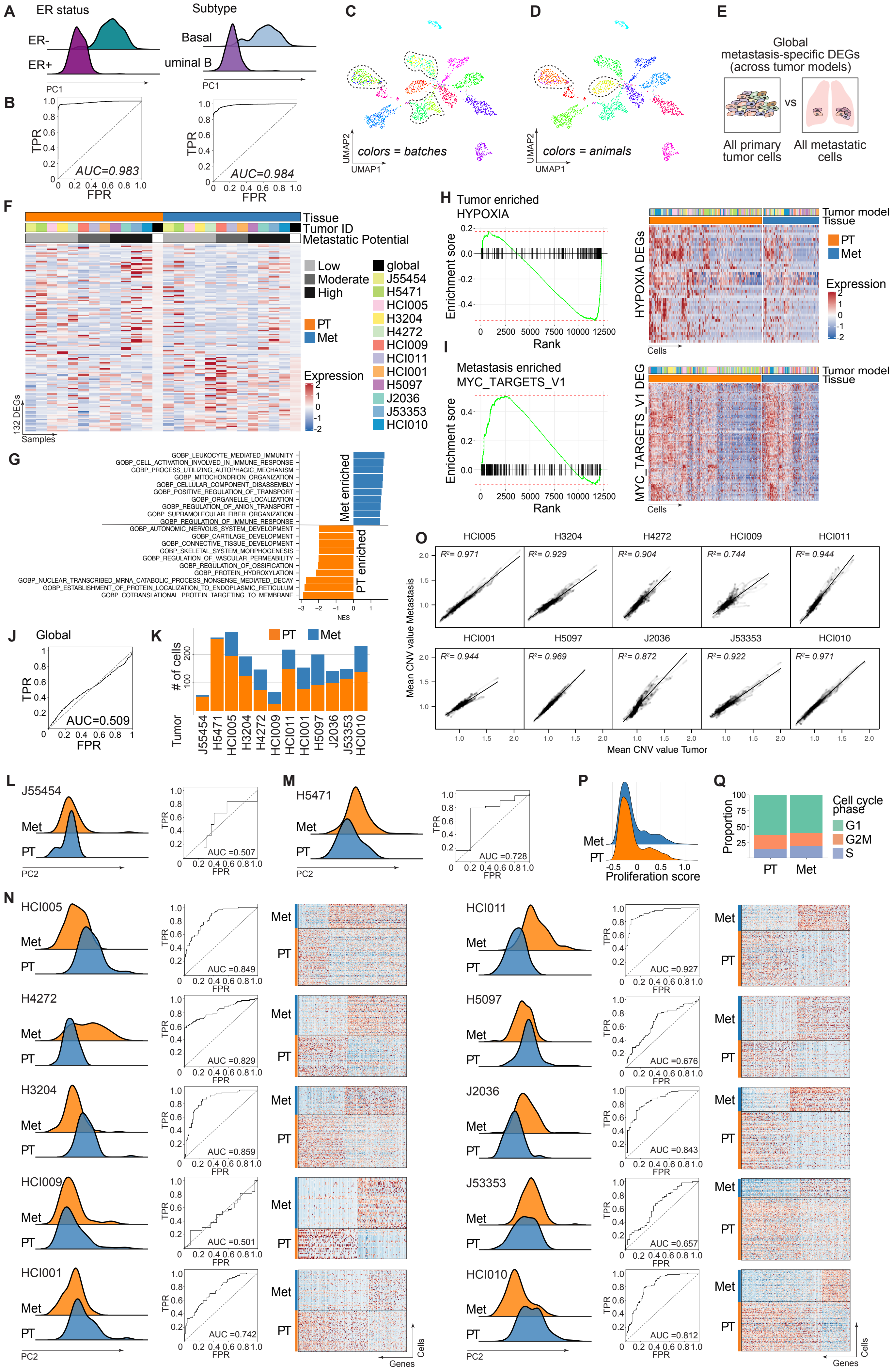
A
ER status
ER-
ER+
PC1
Subtype
Basal
uminal B
PC1
B
TPR
FPR
AUC=0.983
AUC=0.984
C
colors = batches
UMAP2
UMAP1
D
colors = animals
UMAP2
UMAP1
E
Global
metastasis-specific DEGs
(across tumor models)
vs
All primary
tumor cells
All metastatic
cells
F
Tissue
Tumor ID
Metastatic Potential
Low
Moderate
High
PT
Met
Expression
global
J55454
H5471
HCI005
H3204
H4272
HCI009
HCI011
HCI001
H5097
J2036
J53353
HCI010
132 DEGs
Samples
G
GOBP_LEUKOCYTE_MEDIATED_IMMUNITY
GOBP_CELL_ACTIVATION_INVOLVED_IN_IMMUNE_RESPONSE
GOBP_PROCESS_UTILIZING_AUTOPHAGIC_MECHANISM
GOBP_MITOCHONDRION_ORGANIZATION
GOBP_CELLULAR_COMPONENT_DISASSEMBLY
GOBP_POSITIVE_REGULATION_OF_TRANSPORT
GOBP_ORGANELLE_LOCALIZATION
GOBP_REGULATION_OF_ANION_TRANSPORT
GOBP_SUPRAMOLECULAR_FIBER_ORGANIZATION
GOBP_REGULATION_OF_IMMUNE_RESPONSE
GOBP_AUTONOMIC_NERVOUS_SYSTEM_DEVELOPMENT
GOBP_CARTILAGE_DEVELOPMENT
GOBP_CONNECTIVE_TISSUE_DEVELOPMENT
GOBP_SKELETAL_SYSTEM_MORPHOGENESIS
GOBP_REGULATION_OF_VASCULAR_PERMEABILITY
GOBP_REGULATION_OF_OSSIFICATION
GOBP_PROTEIN_HYDROXYLATION
GOBP_NUCLEAR_TRANSCRIBED_MRNA_CATABOLIC_PROCESS_NONSENSE_MEDIATED_DECAY
GOBP_ESTABLISHMENT_OF_PROTEIN_LOCALIZATION_TO_ENDOPLASMIC_RETICULUM
GOBP_COTRANSLATIONAL_PROTEIN_TARGETING_TO_MEMBRANE
Met enriched
PT enriched
NES
H
Tumor enriched
HYPOXIA
Enrichment sore
Rank
HYPOXIA DEGs
Tumor model
Tissue
PT
Met
Expression
Cells
I
Metastasis enriched
MYC_TARGETS_V1
Enrichment sore
Rank
MYC_TARGETS_V1 DEG
Tumor model
Tissue
Cells
J
Global
TPR
FPR
AUC=0.509
K
# of cells
PT
Met
Tumor
J55454
H5471
HCI005
H3204
H4272
HCI009
HCI011
HCI001
H5097
J2036
J53353
HCI010
O
HCI005
H3204
H4272
HCI009
HCI011
R²= 0.971
R²= 0.929
R²= 0.904
R²= 0.744
R²= 0.944
HCI001
H5097
J2036
J53353
HCI010
R²= 0.944
R²= 0.969
R²= 0.872
R²= 0.922
R²= 0.971
Mean CNV value Metastasis
Mean CNV value Tumor
L
J55454
Met
PT
PC2
AUC =0.507
M
H5471
Met
PT
PC2
AUC =0.728
P
Met
PT
Proliferation score
Q
Proportion
Cell cycle
phase
G1
G2M
S
PT
Met
N
HCI005
AUC =0.849
H4272
AUC =0.829
H3204
AUC =0.859
HCI009
AUC =0.501
HCI001
AUC =0.742
HCI011
AUC =0.927
H5097
AUC =0.676
J2036
AUC =0.843
J53353
AUC =0.657
HCI010
AUC =0.812
Met
PT
PC2
TPR
FPR
Cells
Genes

Supplementary Figure 2: Differential gene expression between primary tumor and matched metastatic cells

A) Ridge plot showing the normalized number of cells along Principal Component (PC) 1 coordinates distinguished by ER status (left) and BC subtype (right).
B) ROC curves with the corresponding area under the curve (AUC) showing PC1 distinguishing tumor models based on ER status (left) or BC subtype (right).
C) UMAP projection of single-cell transcriptomes color-coded by batch (individual plates). Dashed lines highlight clusters of cells from the same tumor measured in multiple batches (technical replicates).
D) Same as in C color-coded by individual animals. Dashed lines highlight clusters of cells from the same model retrieved from multiple animals (biological replicates).
E) Workflow for identifying metastasis-specific DEGs across tumors.
F) Heatmap showing the mean expression of DEGs per model between primary tumors and metastases.
G) Bar chart showing pathways enriched in primary tumors (negative NES, orange) and metastases (positive NES, blue) using GO biological pathways from MSigDB.
H) Enrichment plot showing hypoxia as the top enriched pathway in primary tumors (left). Heatmap showing expression of DEGs associated with hypoxia in single cells (right).
I) Same as in H for MYC targets as the top enriched pathway in metastases.
J) ROC curve using PC2 coordinates to classify cells into either primary tumor or metastatic cells of all tumors grouped together (global) with depicted AUC.
K) Bar chart shows the number of primary tumor (PT) and metastatic (Met) cells for each tumor.
L) Ridge plots (left) showing normalized cell counts along PC2 of primary tumor and metastases for model J55454, which lacked a sufficient number of metastatic cells, and corresponding ROC curves (right).
M) Same as in L showing model H5471.
N) Ridge plots showing normalized cell counts along PC2 of primary tumors and metastases for individual tumor models with a sufficient number of metastatic cells and corresponding ROC curves of PC2. The clear separations in PC2 are reflected by an AUC > 0.7 in the ROC curve analysis. Heatmaps showing the expression of DEGs between primary tumor and metastatic cells.
O) Scatter plot showing correlation of mean CNV values per genomic region determined in primary tumor and metastatic cells for each tumor.
P) Ridge plot showing the proliferation score for primary tumors and metastases.
Q) Bar chart showing the proportion of cells in G1/G2M/S cell cycle phase for primary tumors and metastases. The Wilcoxon rank test revealed no significant differences between the two groups.

A

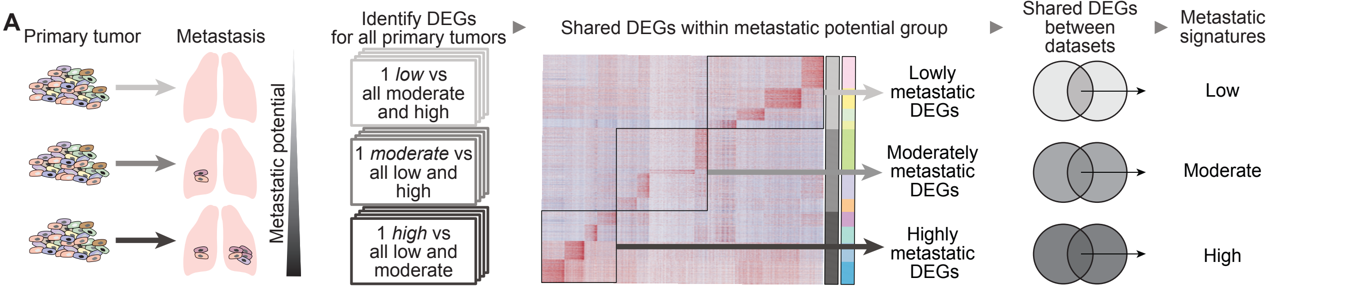

B C D F

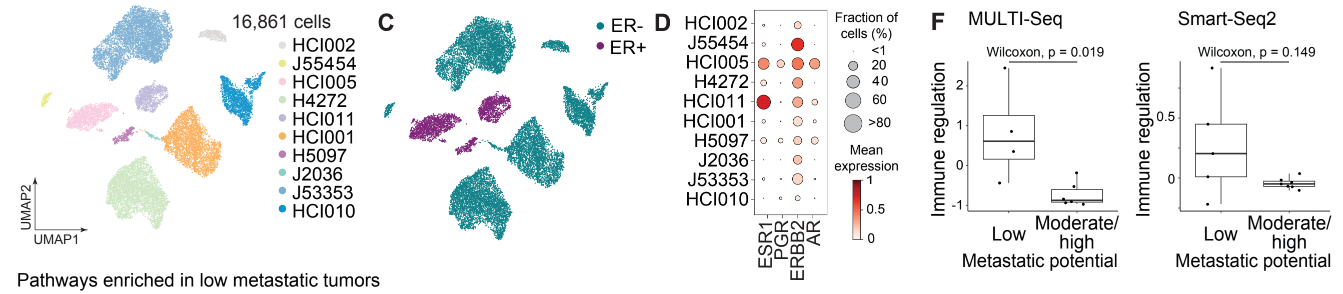

E

Pathways enriched in low metastatic tumors

Pathway (HALLMARK)	p-value	FDR q-value	Genes
TNFA_SIGNALING_VIA_NFKB	6.17E-11	3.09E-09	FOS, CCND1, AREG, ZFP36, TAP1, SOD2, NFKBIA, ATF3, SQSTM1, DUSP1
ESTROGEN_RESPONSE_LATE	1.48E-09	2.46E-08	FOS, CCND1, AREG, ZFP36, KRT19, SLC9A3R1, BLVRB, PLAAT3, CD9
INTERFERON_GAMMA_RESPONSE	1.48E-09	2.46E-08	B2M, UBE2L6, PSMB8, IFI35, HLA-A, HLA-B, TAP1, SOD2, NFKBIA
INTERFERON_ALPHA_RESPONSE	4.00E-09	5.00E-08	B2M, UBE2L6, PSMB8, IFI35, CD47, HLA-C, TAP1
ESTROGEN_RESPONSE_EARLY	3.13E-08	3.13E-07	FOS, CCND1, AREG, KRT19, SLC9A3R1, BLVRB, PLAAT3, ELF3

Pathway (GOBP)	p-value	FDR q-value	Genes
REGULATION_OF_CELL_POPULATION_PRO LIFERATION	2.63E-12	1.67E-08	HLA-E, HLA-A, B2M, ZFP36, NFKBIA, SOD2, ROMO1, LGALS3, CD47, IFI35, CDK6, DUSP1, ATF3, SLC9A3R1, CD9, NUPR1, MALAT1, CCND1, S100A11, PLAAT4, AREG, FTH1, RARRES1 CAPN1
ANTIGEN_PROCESSING_AND_PRESENTATI ON_OF_ENDOGENOUS_PEPTIDE_ANTIGEN	4.36E-12	1.67E-08	HLA-E, HLA-A, B2M, HLA-B, HLA-C, TAP1
ANTIGEN_PROCESSING_AND_PRESENTATI ON_OF_ENDOGENOUS_ANTIGEN	3.65E-11	8.66E-08	HLA-E, HLA-A, B2M, HLA-B, HLA-C, TAP1
BIOLOGICAL_PROCESS_INVOLVED_IN_INTE RSPECIES_INTERACTION_BETWEEN_ORGA	4.52E-11	8.66E-08	HLA-E, HLA-A, B2M, ZFP36, NFKBIA, SOD2, ROMO1, LGALS3, CD47, IFI35, CDK6, HLA-B, HLA-C, OPTN, FOS, S100A14, WFDC2, PLAAT3, OCIAD2, C15orf48, IFI6, CHMP2A
ANTIGEN_PROCESSING_AND_PRESENTATI ON_OF_PEPTIDE_ANTIGEN_VIA_MHC_CLAS	7.48E-11	1.15E-07	HLA-E, HLA-A, B2M, HLA-B, HLA-C, TAP1

Pathway (Reactome)	p-value	FDR q-value	Genes
INNATE_IMMUNE_SYSTEM	1.66E-16	2.68E-13	HLA-A, HLA-B, HLA-C, B2M, LAMTOR2, LAMTOR1, LAMP2, LGALS3, DSP, CAPN1, FTH1, CD59, CD47, MGST1, S100A11, CREG1, HLA-E, PSMB8, UBC, PSMB3, FOS, UBE2L6, NFKBIA, ATP6V0E1
NEUTROPHIL_DEGRANULATION	4.01E-14	3.24E-11	HLA-A, HLA-B, HLA-C, B2M, LAMTOR2, LAMTOR1, LAMP2, LGALS3, DSP, CAPN1, FTH1, CD59, CD47, MGST1, S100A11, CREG1
CYTOKINE_SIGNALING_IN_IMMUNE_SYSTEM	1.54E-12	8.31E-10	HLA-A, HLA-B, HLA-C, B2M, HLA-E, PSMB8, UBC, PSMB3, FOS, UBE2L6, NFKBIA, SOD2, IFI6, IFI35, SQSTM1, CCND1, IL32
ANTIGEN_PROCESSING_CROSS_PRESENTA TION	4.97E-12	2.01E-09	HLA-A, HLA-B, HLA-C, B2M, HLA-E, PSMB8, UBC, PSMB3, TAP1
ANTIGEN_PRESENTATION_FOLDING_ASSEM BLY_AND_PEPTIDE_LOADING_OF_CLASS_I_	2.81E-11	8.88E-09	HLA-A, HLA-B, HLA-C, B2M, HLA-E, TAP1

H

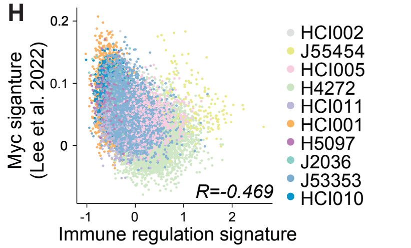

G

Pathways enriched in high metastatic tumors

Pathway (HALLMARK)	p-value	FDR q-value	Genes
OXIDATIVE_PHOSPHORYLATION	5.57E-11	2.78E-09	CYC1, MDH2, GPX4, ECHS1, MRPL15, NDUFB7, UQCRC1, LDHB, IDH2, UQCRFS1
MYC_TARGETS_V1	1.35E-09	3.37E-08	CYC1, MDH2, RACK1, PRDX4, SRM, FBL, RPS2, RPS3, RPS6, RPL14
ADIPOGENESIS	5.43E-07	6.79E-06	CYC1, MDH2, GPX4, ECHS1, MRPL15, NDUFB7, UQCRC1
EPITHELIAL_MESENCHYMAL_TRANSITION	5.43E-07	6.79E-06	TPM2, BGN, GJA1, EMP3, CTHRC1, PCOLCE, VIM
MYOGENESIS	8.79E-06	7.33E-05	TPM2, SPHK1, CLU, CFD, APOD, IGFBP7

Pathway (Reactome)	p-value	FDR q-value	Genes
TRANSLATION	3.95E-14	2.87E-10	RPS2, RPS6, RPS3, RPS3A, RPS20, RPL7, RPL13, RPL32, RPL37, RPL14, RPN2, TRAM1, EIF3E, EIF3H, EEF1D, MRPL15
SRP_DEPENDENT_COTRANSLATIONAL_PRO TEIN_TARGETING_TO_MEMBRANE	7.50E-14	2.87E-10	RPS2, RPS6, RPS3, RPS3A, RPS20, RPL7, RPL13, RPL32, RPL37, RPL14, RPN2, TRAM1
EUKARYOTIC_TRANSLATION_INITIATION	5.16E-13	1.32E-09	RPS2, RPS6, RPS3, RPS3A, RPS20, RPL7, RPL13, RPL32, RPL37, RPL14, EIF3E, EIF3H
EUKARYOTIC_TRANSLATION_ELONGATION	2.38E-11	3.95E-08	RPS2, RPS6, RPS3, RPS3A, RPS20, RPL7, RPL13, RPL32, RPL37, RPL14, EEF1D
CELLULAR_RESPONSES_TO_STIMULI	2.58E-11	3.95E-08	RPS2, RPS6, RPS3, RPS3A, RPS20, RPL7, RPL13, RPL32, RPL37, RPL14, TUBA1B, CYBA, HMGA1, CDKN2A, IGFBP7, UBE2S, PRDX2, BLVRB, MYDGF,

Pathway (GOBP)	p-value	FDR q-value	Genes
PEPTIDE_METABOLIC_PROCESS	3.95E-14	2.87E-10	PKM, RPS3, RPL37, RPS20, RPS6, RPS3A, RPS2, RPL7, RPL14, RPL13, RPL32, RACK1, VIM, EEF1D, EIF3E, EIF3H, CLU, GPX4, TMED10, ETHE1
CYTOPLASMIC_TRANSLATION	7.50E-14	2.87E-10	PKM, RPS3, RPL37, RPS20, RPS6, RPS3A, RPS2, RPL7, RPL14, RPL13, RPL32
CELLULAR_AMIDE_METABOLIC_PROCESS	5.16E-13	1.32E-09	PKM, RPS3, RPL37, RPS20, RPS6, RPS3A, RPS2, RPL7, RPL14, RPL13, RPL32, RACK1, VIM, EEF1D, EIF3E, EIF3H, CLU, GPX4, TMED10, ETHE1, SPHK1
PEPTIDE_BIOSYNTHETIC_PROCESS	2.38E-11	3.95E-08	PKM, RPS3, RPL37, RPS20, RPS6, RPS3A, RPS2, RPL7, RPL14, RPL13, RPL32, RACK1, VIM, EEF1D, EIF3E, EIF3H
AMIDE_BIOSYNTHETIC_PROCESS	2.58E-11	3.95E-08	PKM, RPS3, RPL37, RPS20, RPS6, RPS3A, RPS2, RPL7, RPL14, RPL13, RPL32, RACK1, VIM, EEF1D, EIF3E, EIF3H

I

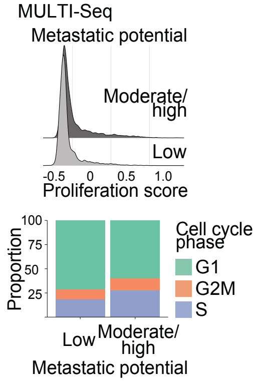

J

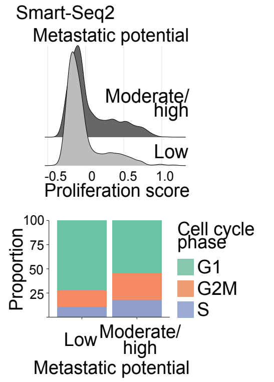

Supplementary Figure 3: Characteristics of the metastatic signatures

A) Schematic workflow for the identification of metastatic signatures.
B) UMAP projection of single-cell transcriptomes color-coded by individual tumors.
C) Same as in B color-coded by ER status.
D) Bubble plot showing the gene expression of receptors per tumor.
E) Enriched pathways identified using the DEGs shared among poorly metastatic tumors.
F) Boxplot showing immune regulation signature expression comparing low and moderate/high metastatic potential tumor models. Significance (p value) is indicated using the Wilcoxon rank test.
G) Enriched pathways identified using the DEGs shared among highly metastatic tumors.
H) Scatterplot showing the correlation of MYC[41] and immune regulation signature expression. The Pearson correlation coefficient is shown.
I) Ridge plot showing proliferation score for primary tumors with low and moderate/high metastatic potential. Bar chart showing the proportion of cells in different cell cycle phases for primary tumors of low and moderate/high metastatic potential. The MULTI-seq dataset is shown. The Wilcoxon rank test revealed no significant changes in proportions.
J) Same as in I for the Smart-Seq2 dataset. The Wilcoxon rank test revealed no significant differences.

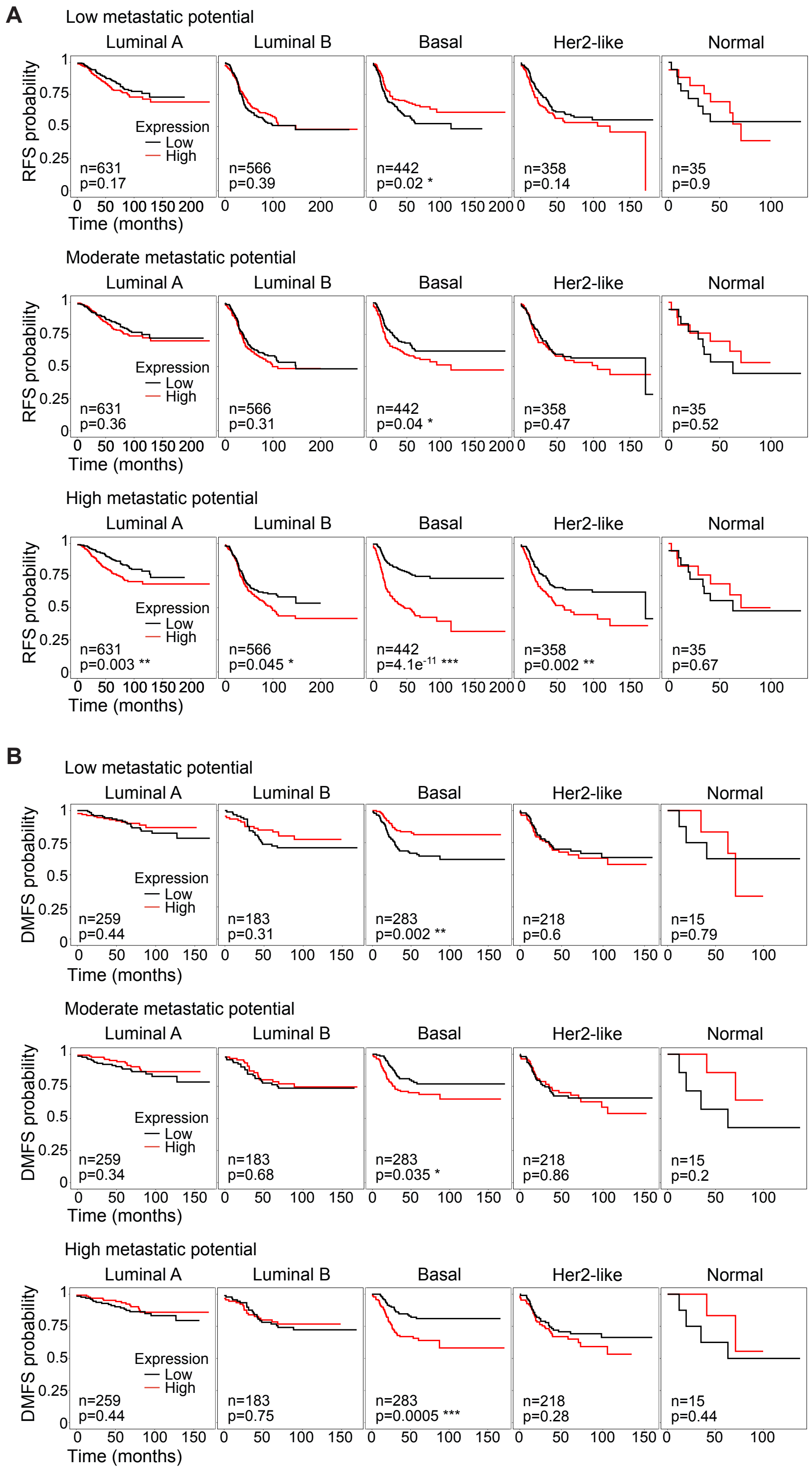
A
Low metastatic potential
Moderate metastatic potential
High metastatic potential
Luminal A
Luminal B
Basal
Her2-like
Normal
RFS probability
Time (months)
Expression
Low
High
n=631
p=0.17
n=566
p=0.39
n=442
p=0.02 *
n=358
p=0.14
n=35
p=0.9
n=631
p=0.36
n=566
p=0.31
n=442
p=0.04 *
n=358
p=0.47
n=35
p=0.52
n=631
p=0.003 **
n=566
p=0.045 *
n=442
p=4.1e-11 ***
n=358
p=0.002 **
n=35
p=0.67
B
Low metastatic potential
Moderate metastatic potential
High metastatic potential
DMFS probability
n=259
p=0.44
n=183
p=0.31
n=283
p=0.002 **
n=218
p=0.6
n=15
p=0.79
n=259
p=0.34
n=183
p=0.68
n=283
p=0.035 *
n=218
p=0.86
n=15
p=0.2
n=259
p=0.44
n=183
p=0.75
n=283
p=0.0005 ***
n=218
p=0.28
n=15
p=0.44

Supplementary Figure 4: Metastatic signatures are associated with poor patient-related outcomes

A) Kaplan-Meier-plots showing the Recurrence-free survival (RFS) of BC patients (generated with KM-plotter[42]) stratified by PAM50 BC subtype using the mean expression of the low metastatic potential (top panel), moderate metastatic potential EMP (middle panel), and high metastatic potential signatures (lower panel). The number of patients (n) and p values (p) are shown calculated with the log-rank test. * indicates significance.

B) Same as in A) showing the distant metastasis-free survival (DMFS).

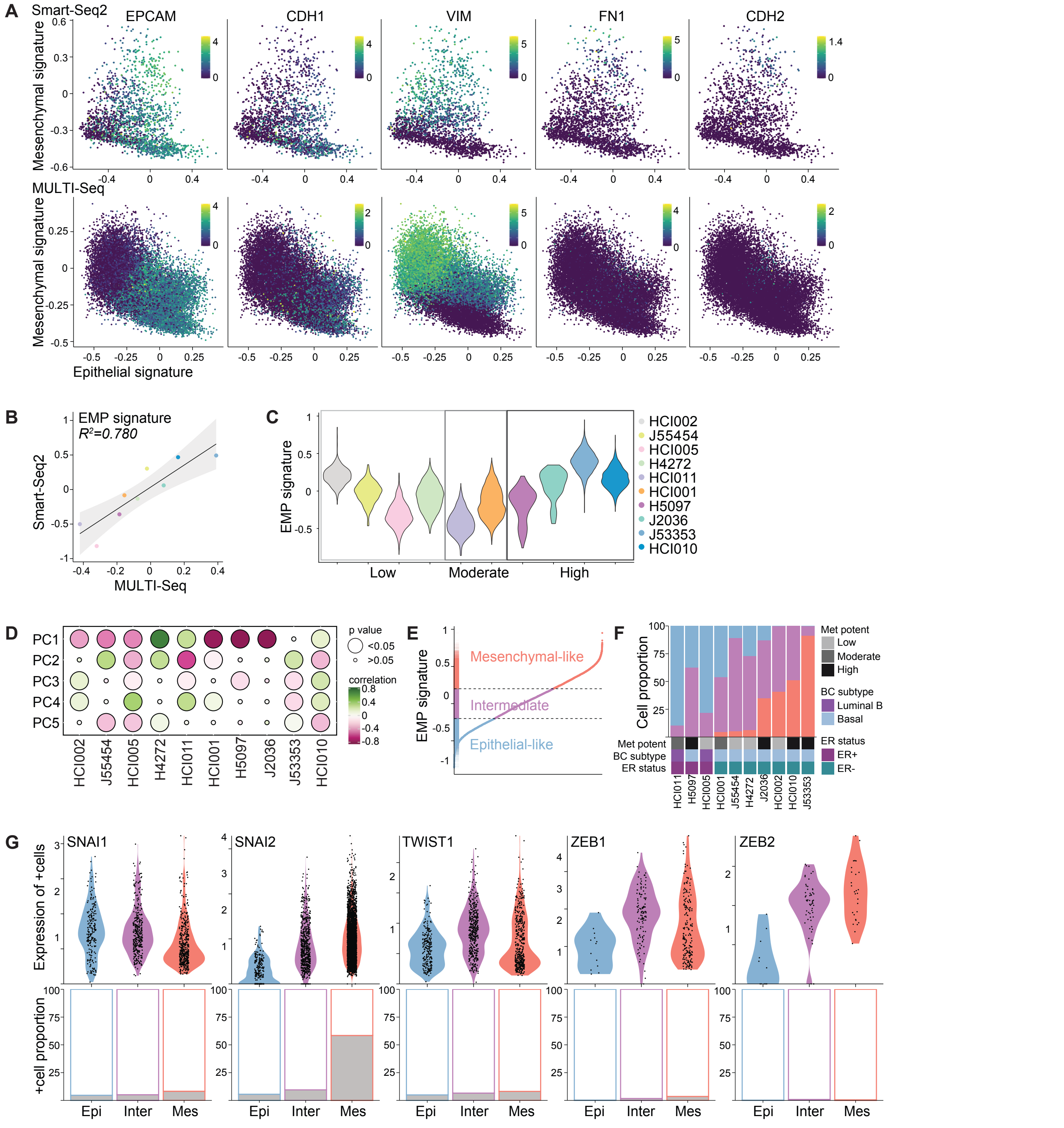
A
Smart-Seq2
EPCAM
CDH1
VIM
FN1
CDH2
Mesenchymal signature
MULTI-Seq
Epithelial signature
B
EMP signature
R^2=0.780
Smart-Seq2
MULTI-Seq
C
EMP signature
Low
Moderate
High
HCI002
J55454
HCI005
H4272
HCI011
HCI001
H5097
J2036
J53353
HCI010
D
PC1
PC2
PC3
PC4
PC5
p value
<0.05
>0.05
correlation
E
EMP signature
Mesenchymal-like
Intermediate
Epithelial-like
F
Cell proportion
Met potent
Low
Moderate
High
BC subtype
Luminal B
Basal
ER status
ER+
ER-
G
SNAI1
SNAI2
TWIST1
ZEB1
ZEB2
Expression of +cells
+cell proportion
Epi
Inter
Mes

Supplementary Figure 5: EMP is a key feature of tumor heterogeneity

A) Scatter plots showing mesenchymal versus epithelial signatures for individual cells colored by the gene expression of epithelial (EPCAM, CDH1) and mesenchymal markers (VIM, FN1, CDH2) colored by magnitude of scaled gene expression. Showing Smart-Seq2 (upper panel) and MULTI-seq datasets (lower panel).

B) Scatter plot showing the correlation of the mean EMP signature expression per tumor between the Smart-Seq2 and MULTI-seq datasets. Linear regression with 95% confidence intervals and Pearson correlation coefficient are shown.

C) Violin plot showing EMP signature expression per tumor ordered by metastatic potential using the MULTI-seq dataset.

D) Bubble plot showing the correlation of the EMP signature with PCs 1-5 using the MULTI-seq dataset.

E) Cells ranked by EMP signature defining three cell states: epithelial-like (blue), intermediate EMP (purple) and mesenchymal-like cells (red) using the MULTI-seq dataset.

F) Bar chart showing the proportion of EMP cell states per tumor ranked by the increasing proportion of mesenchymal-like cells. The MULTI-seq dataset is shown.

G) Violin plots (top) showing expression of EMT-associated TFs in expressing cells grouped by EMP cell states (Epi = epithelial-like, Inter = Intermediate EMP, Mes = mesenchymal-like cells). Bar charts (bottom) showing the fraction of expressing cells in gray. The MULTI-seq dataset is shown.

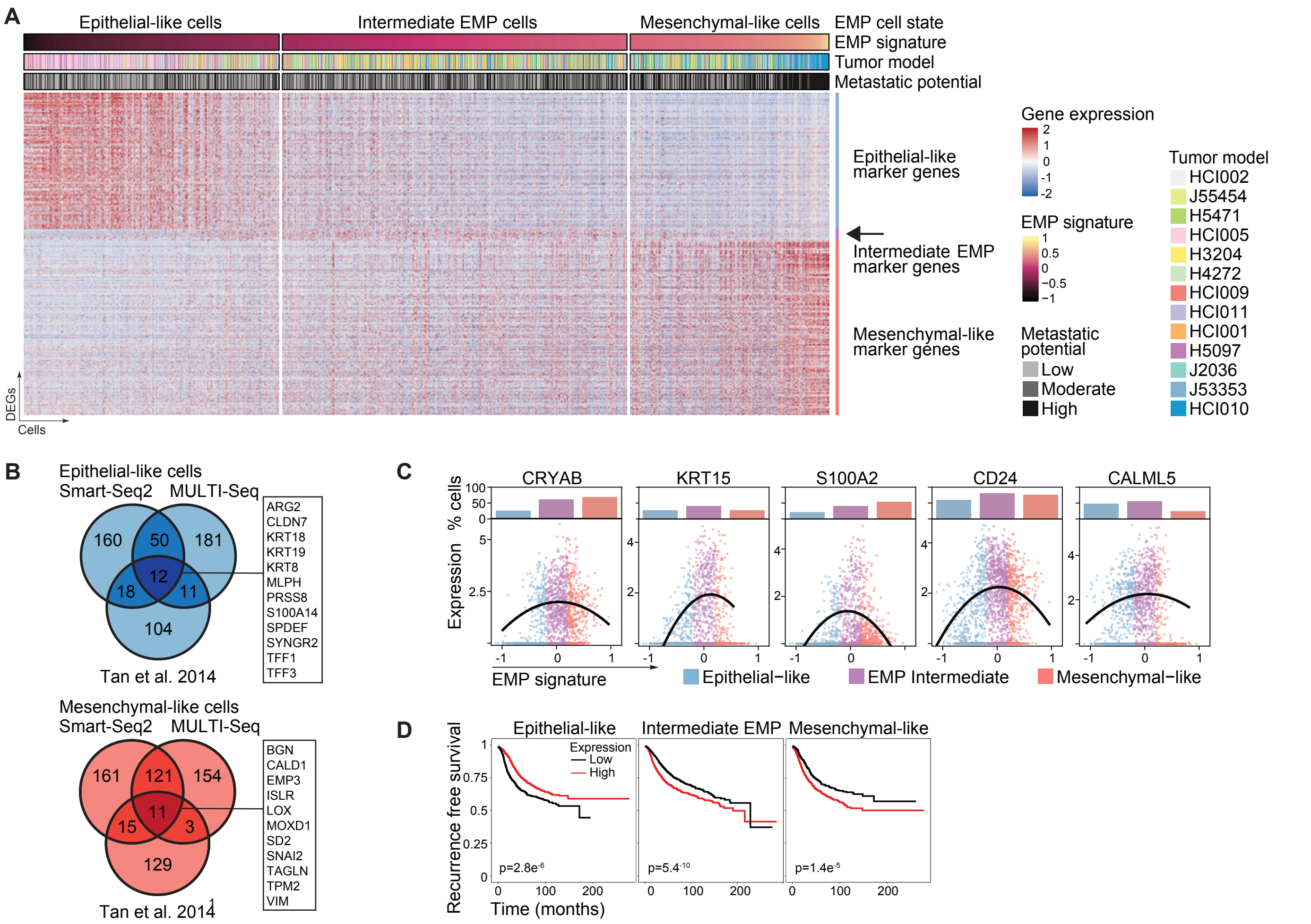
A
Epithelial-like cells
Intermediate EMP cells
Mesenchymal-like cells
EMP cell state
EMP signature
Tumor model
Metastatic potential
Epithelial-like marker genes
Intermediate EMP marker genes
Mesenchymal-like marker genes
Gene expression
EMP signature
Metastatic potential
Low
Moderate
High
Tumor model
HCI002
J55454
H5471
HCI005
H3204
H4272
HCI009
HCI011
HCI001
H5097
J2036
J53353
HCI010
DEGs
Cells
B
Epithelial-like cells
Smart-Seq2
MULTI-Seq
Tan et al. 2014
ARG2
CLDN7
KRT18
KRT19
KRT8
MLPH
PRSS8
S100A14
SPDEF
SYNGR2
TFF1
TFF3
Mesenchymal-like cells
BGN
CALD1
EMP3
ISLR
LOX
MOXD1
SD2
SNAI2
TAGLN
TPM2
VIM
C
CRYAB
KRT15
S100A2
CD24
CALML5
% cells
Expression
EMP signature
Epithelial−like
EMP Intermediate
Mesenchymal−like
D
Epithelial-like
Intermediate EMP
Mesenchymal-like
Expression
Low
High
Recurrence free survival
Time (months)
p=2.8e-6
p=5.4-10
p=1.4e-5

Supplementary Figure 6: Intermediate EMP cells are characterized by specific markers

A) Heatmap showing DEGs for epithelial-like, mesenchymal-like, and intermediate EMP cells from the Smart-Seq2 data. Cells are ordered by increasing EMP signature. Annotations indicate the EMP cell state, EMP signature expression, tumor model and metastatic potential. The arrow highlights intermediate EMP cell marker genes.
B) Venn diagrams showing the number of overlapping epithelial (blue, left panel) and mesenchymal markers (red, right panel) obtained from Smart-Seq2, MULTI-seq and Tan et al. 2014. Genes shared among all three sets are highlighted.
C) Scatter plots showing the expression of the indicated genes ordered by increasing EMP signature expression. The dots show the expression in individual cells, and lines show smoothed expression in expressing cells. The bar charts on top show the proportion of positive expressing cells for the EMP cell states. The Smart-Seq2 dataset is shown.
D) Kaplan-Meier-plots showing the RFS of BC patients stratified using the mean expression of the overlapping genes for each EMP cell state (generated with KM-plotter[42]). p values were calculated using the log-rank test.
